# Do women's empowerment and self-expression values change adolescents' gendered occupational expectations? Longitudinal evidence against the gender-equality paradox from 26 European countries

**DOI:** 10.3389/fsoc.2023.1175651

**Published:** 2023-06-09

**Authors:** Melinda Erdmann, Agustina Marques Hill, Marcel Helbig, Kathrin Leuze

**Affiliations:** ^1^President's Research Group, Berlin Social Science Center, Berlin, Germany; ^2^Skill Formation and Labor Markets, Berlin Social Science Center, Berlin, Germany; ^3^Department of Educational Decisions and Processes, Migration, Returns to Education, Leibniz Institute for Educational Trajectories, Bamberg, Germany; ^4^Institute of Sociology, Friedrich Schiller University of Jena, Jena, Germany

**Keywords:** gender occupational expectations, PISA, gender-equality paradox, gender norms, self-expression values, women's empowerment

## Abstract

Despite the increases in women's educational attainment in recent decades, female labor market participation and labor market returns are still lower than those of their male counterparts. Among the main factors explaining this persistence of economic inequality is the persistently gendered nature of occupational expectations, which results in gender segregation of labor. In this paper, we describe how gender-specific adolescents' occupational expectations change over time (2006–2018) and how women's empowerment and cultural norms might influence gender-specific occupational expectations. Against the backdrop of the research on the gender-equality paradox and from a comparative perspective, we focus on national and institutional characteristics to investigate how individual and national factors explain gendered occupational expectations. We answer our research questions by applying a two-step multilevel model with fixed effects. For this, we used PISA data and merged them with state-level information from 26 European countries. We add to existing research by making three contributions. First, we describe the changes in occupational expectations over time within European countries by looking at the gender composition of the desired occupation and distinguishing three categories (gender-typical, gender-balanced, and gender-atypical). Second, we investigate the relationship between national characteristics and the evolution of gendered occupational expectations separately by gender to reveal gender-specific mechanisms. Third, by using data from two-time points, we explore which national-level changes lead to changes in students' occupational expectations. Our first descriptive results show that the patterns of how students' occupational expectations change over time differ remarkably between countries. In 2018 in some countries, students' occupational expectations became more segregated while in others the number of students with gender-balanced or gender-atypical expectations increased. Our fixed effects models show that women's empowerment and self-expression value explained variance over time. For example, women's empowerment measured via an increase in women's employment and participation in parliament led to less gender-typical occupational expectations among girls and boys. Similarly, a rise in self-expression values led to less gender-typical occupational expectations, again for both boys and girls. Remarkably, our results do not verify the gender-equality paradox for occupational expectations, as is the case in previous cross-sectional analyses.

## 1. Introduction

Despite women's increasing educational attainment over the last decades, women's labor market participation and returns are still lower than those of men. One of the main explanatory factors for this is the persistence of gendered occupational expectations among young people, which results in a gender-segregated structure of the labor market. Even though occupational gender segregation is found today in all OECD countries (Anker, 1998), its scope varies considerably across countries (Jarman et al., 2012). Remarkably, gender segregation in the labor market is most pronounced in advanced industrialized countries (Charles and Grusky, 2004; Jarman et al., 2012). For example, occupational gender segregation is highest in countries like Denmark, Finland, and Sweden, which are viewed as the most culturally progressive and economically advanced (Jarman et al., 2012). Based on modernization theory, researchers expected the opposite: societies with higher women's empowerment and participation and more prevalent egalitarian values were expected to show a more gender-balanced occupational distribution (for a discussion, see Charles, 2011). Therefore, the prevailing cross-national pattern of occupational gender segregation is counterintuitive for many researchers. It is therefore called the “gender-equality paradox” (Charles, 2011; Stoet and Geary, 2018).

This paradox is not limited to the development of gender segregation in the labor market: it starts in secondary education when adolescents develop their occupational preferences. For example, more progressive countries exhibit larger gender gaps in science self-concepts (Sikora and Pokropek, 2012), math attitudes (Charles et al., 2014; Breda et al., 2020), math anxiety (Stoet et al., 2016), aspirations and expectations for certain college majors (for STEM, Kjærnsli and Lie, 2011; McDaniel, 2016), and occupational aspirations (Charles, 2017; Barrett, 2021; Basler et al., 2021; Stoet and Geary, 2022). In this paper, we contribute to this literature by examining cross-national variations in the development of gendered occupational expectations among young people over time to further our understanding of occupational gender segregation in the labor market. Thus, we answer the question of how occupational expectations have changed in Europe in the last decade and whether institutional and cultural characteristics can explain these changes.

From a life course perspective, young people's gendered occupational expectations (also called realistic aspirations or plans) are the starting point for a long-lasting process of occupational preference formation that ultimately results in gendered occupational choices in the labor market (e.g., Morgan et al., 2013; Polavieja and Platt, 2014; Law, 2018). Thus, by examining changes in adolescents' occupational expectations, we can further our understanding of the evolution of gender segregation in the labor market and the roots of the gender-equality paradox. Recent research on gendered occupational expectations either investigated single countries, giving important insights on individual and social factors affecting gendered occupational expectations (e.g., Barrett, 2021; Basler et al., 2021), or conducted country comparisons with cross-sectional data, providing initial evidence on institutional and cultural features shaping this process (e.g., Sikora and Saha, 2009; Han, 2015; Hillmert, 2015; Mann and DiPrete, 2016; Hägglund and Leuze, 2021).

In particular, education system features (e.g., Hillmert, 2015; Blasko et al., 2018), labor market structures (e.g., Hägglund and Leuze, 2021), and cultural gender norms and values (e.g., Leuze and Helbig, 2015) have been identified as relevant factors. Given the counterintuitive pattern of the gender-equality paradox, researchers either investigated how women's empowerment and participation in different societal domains or how cultural norms and values shape young peoples' occupational aspirations. However, such results come from cross-sectional studies that investigate between-country variations. These do not allow for causal assessments. By contrast, studies that investigate within-country variation as changes over time would allow for more causal empirical assessments. In this regard, research on gender differences in personality traits has already shown that with a longitudinal perspective, the pattern of the gender-equality paradox is not observable (see Fors Connolly et al., 2020). Therefore, we extend previous research by combining a comparative and a longitudinal perspective on the development of gendered occupational expectations to reassess the explanatory factors of the gender-equality paradox. More precisely, we investigate how young people's gendered occupational expectations changed between 2006 and 2018 in 26 European countries and whether women's empowerment and/or gender norms and values can explain these changes.

By adopting this approach, we make three contributions to previous research. First, we describe the changes in occupational expectations over time within European countries by looking at the gender composition of occupations. The two existing comparative studies investigating changes looked at STEM (Science, Technics, Engineering, and Mathematics) expectations (Charles, 2017; Blasko et al., 2018) rather than changes in the gender-typing of occupations in general. By illustrating the changes in the gender composition of occupations and distinguishing three categories (gender-typical, gender-balanced, and gender-atypical), we can observe a more complex developmental pattern in occupational expectations rather than only that for specific occupations, as in the case of STEM. This allows us to show whether occupational expectations have moved more in the direction of gender-typical or gender-atypical expectations, or whether they moved more in the direction of gender-balanced expectations. Second, we investigate the relationship between national characteristics and the evolution of gendered occupational expectations separately by gender. Earlier research has shown that young women and men are affected differently by the process of modernization regarding their occupational expectations (Hägglund and Leuze, 2021), which is obscured when looking at the overall development of gender segregation. Our gender-differentiated analyses thus enable us to identify gender-specific mechanisms contributing to the gender-equality paradox. Third, we use data from two time-points, the 2006 and 2018 Programme of International Student Assessment (PISA), to explore which changes on the national level led to changes in students' occupational expectations. In contrast to previous cross-sectional approaches, we thus exploit variations over time within countries and apply multilevel panel regression models to control for time-invariant, unobserved national factors that might additionally influence occupational expectations and are not accounted for by cross-sectional designs.

Our article is structured as follows. After presenting the state of research, we discuss the theoretical assumption behind the gender-equality paradox and how individual characteristics, institutional characteristics, and cultural values might affect changes in gendered occupational expectations. After a description of the different data sources and our methodological strategy, we present the results for the development of gendered occupational expectations over time and for the factors that influence young people's occupational expectations on the national level. We conclude with suggestions for further research and policies and discuss the methodological limitations of our analysis.

## 2. State of research

Occupational expectations (also called realistic aspirations or plans) are conceptualized as realistic appraisals of occupational attainment based on actual or perceived opportunities, barriers, and constraints (Kerckhoff, 1976; Gottfredson, 1981). There is a wide range of research on gender-specific occupational expectations. This research includes different focuses and different methodological approaches. Some studies examine individual and social factors to explain variation in gender-specific occupational expectations within a single country (e.g., Malin and Jacob, 2019; Barrett, 2021)^1^; others scrutinize variations across countries and country-level explanations (e.g., Sikora and Pokropek, 2012; Leuze and Helbig, 2015; Stoet and Geary, 2022). Most of this comparative research has focused on gendered preferences for specific fields of occupations, for example, STEM occupations (Blasko et al., 2018; Hägglund and Leuze, 2021) or person-and thing-orientated occupations (Stoet and Geary, 2022), while only very few studies examined the gender composition of occupational expectations (Hillmert, 2015; Leuze and Helbig, 2015). However, studies that examine gender differences in STEM expectations mainly measure girls' gender-atypical expectations, whereas boys' gender-atypical expectations are mostly not captured.^2^ Therefore, we investigate the gender-typing of occupations in general by measuring the share of women in each occupational category, which allows us to distinguish between male-dominated, female-dominated, and gender-balanced occupations. Only through this approach are we able to analyze whether both girls' and boys' occupational expectations have become more gender-typical or gender-atypical over time. Previous research has shown that young women and men are affected differently by the processes of modernization regarding their occupational expectations (Hägglund and Leuze, 2021). Thus, our gender-differentiated analyses enable us to identify gender-specific mechanisms contributing to the gender-equality paradox.

Previous cross-national comparative studies identified a large variety of institutional and cultural factors at the country level affecting adolescents' occupational preferences. They showed that education system features (Han, 2015; Hillmert, 2015; Blasko et al., 2018), women's participation in different societal spheres (Charles, 2017; Stoet and Geary, 2022), changing labor market structures (Sikora and Saha, 2009; Hägglund and Leuze, 2021), economic development (Charles, 2017); gender stereotypes and societal values (Leuze and Helbig, 2015; Charles, 2017) explain cross-national variations in gendered occupational aspirations and expectations. To explain the gender-equality paradox from a comparative perspective, research focused, on the one hand, on factors that indicate gender-progressive societies and therefore on the empowerment of women and, on the other hand, on the influence of gender norms and values to explain the counterintuitive pattern of young people's occupational expectations.

Regarding the effect of women's empowerment, Hägglund and Leuze (2021) showed that the gender gap in STEM expectations was larger in countries with higher female labor force participation and more women working in management. Similarly, a higher female participation in management (Leuze and Helbig, 2015) and in the labor market (Hillmert, 2015) had a positive effect on gender-typical occupational expectations, especially for girls. Likewise, Stoet and Geary (2022) found larger gender differences in preferences between people-oriented and thing-oriented occupations in countries with a higher level of women's empowerment, as measured by the global gender gap index (GGGI). Only Charles (2017) found an effect that was not in line with the pattern of the gender-equality paradox. In her study, women's labor market participation was positively associated with girls' expectations of math-intensive occupations. Accordingly, most empirical findings on the relationship between women's empowerment and gendered occupational expectations do not support the theoretical propositions derived from modernization approaches, but are rather in line with the gender-equality paradox. Therefore, we aim to reassess these explanatory factors by employing a longitudinal approach.

Even though many scholars conclude that gender norms and gendered self-expression values explain the persistence of gender segregation (Correll, 2004; Charles and Bradley, 2009; England, 2010; Cotter et al., 2011; Cech, 2013) and the gender-equality paradox, only very little comparative research has investigated the meaning of gender stereotypes in modern societies for young people's occupational aspirations. In this context, Leuze and Helbig (2015) found that in countries with stronger self-expressive values, only boys express more gender-typical occupational expectations, whereas girls' expectations tend to be more atypical. Charles (2017) did not find a significant effect of gender stereotypes on STEM occupational expectations, even though her results show more pronounced gender-typical occupational expectations in countries with a higher Human Development Index (HDI).

Most of these comparative studies used cross-sectional institutional and cultural variations across countries to explain gendered occupational expectations. However, cross-sectional designs only allow us to descriptively investigate associations between country-level predictors and individual outcomes. The only two studies we are aware of that looked at changes in occupational expectations over time are Charles (2017) and Blasko et al. (2018), who focused on occupational aspirations to STEM fields. While Blasko et al. (2018) did not find any effect of country characteristics on changing STEM aspirations, Charles (2017) demonstrated that in countries with a higher Human Development Index (HDI), occupational aspirations became more gender-typical.

Summing up, comparative results on the influence of women's empowerment and gender norms and values on young people's occupation expectations are mixed. One the one hand, findings from cross-sectional studies are mostly in line with the pattern of the gender-equality paradox. On the other hand, the limited results of longitudinal studies on STEM expectations hardly support it. Therefore, we aim to reassess the explanatory factors related to the gender-equality paradox with a longitudinal approach on gendered occupational aspirations to overcome the methodological pitfalls of cross-sectional analyses.

In the following, we introduce the theoretical discussion on the gender-equality paradox at the macro level before discussing possible mechanisms at the micro level for understanding how institutional and cultural factors might shape gendered occupational expectations.

## 3. Theoretical considerations: the gender-equality paradox

Evolutionary accounts of the development of occupational gender segregation assume that in the course of modernization, all forms of societal gender inequalities are likely to disappear. This assumption is derived from two arguments that draw on different theories of evolutionary change (for an overview, see Charles, 2011). First, from an economic perspective, excluding women through discrimination is too costly for modern societies due to increasing market competition. Therefore, gender inequalities in all societal spheres, including occupational gender segregation, are assumed to diminish (e.g., Jackson, 1998). Second, from a neo-intuitionalist perspective, egalitarian norms are expected to spread through the internationalization of markets, social movements, and organizations. Furthermore, legal equality, access to the education system, and the labor market have long-term culture-changing effects (e.g., Ramirez, 1997). In more modern countries, stereotypical gendered expectations of the social environment should therefore be less legitimate, since the prevailing gender ideology is generally more egalitarian (Ramirez and Wotipka, 2001; Inglehart, 2008). “By these accounts, sex segregation is a traditional relict that will decline […] as egalitarian values become manifest in attitudes and career aspirations” (Charles and Bradley, 2009, p. 925).

Following these evolutionary accounts, societies with higher female empowerment and participation and more prevalent egalitarian values were expected to show a more gender-balanced occupational distribution (e.g., Ramirez, 1997). Empirically, research initially supported these assumptions by showing a substantial reduction of gender segregation in developed countries over the last 60 years (England and Li, 2006). Nevertheless, more recent studies indicate that this trend slowed down over the last decades and even stopped in the 2000's (England and Li, 2006; Tomaskovic-Devey et al., 2006; Blau et al., 2013). Although women's participation has increased enormously in recent decades, for example in education and the labor market, large gender gaps continued to emerge in some areas and aspects of societies. In the labor market in particular, women have access to higher positions, but this has not simultaneously led to lower overall occupational gender segregation (Jarman et al., 2012).

Although this empowerment of women theoretically widened the range of career options for women in several ways, most subsequent research has shown that especially horizontal gender segregation in occupational aspirations or expectations has remained or even increased in the last decades (Charles and Bradley, 2002). Moreover, it is remarkable that gender segregation in the labor market is most pronounced in most advanced industrialized countries (Jarman et al., 2012) or countries with progressive welfare states (Mandel and Semyonov, 2006). For example, horizontal gender segregation is highest in countries like Denmark, Finland, and Sweden, which are viewed as the most culturally progressive and economically advanced (Jarman et al., 2012). Therefore, the more recent cross-national pattern of occupational gender segregation is “counterintuitive” for many researchers and therefore named the “gender-equality paradox” (Charles, 2011; Stoet and Geary, 2018).

Given the empirically observed pattern and a more revised perspective on the evolution of gender segregation, researchers stressed the importance of distinguishing different types of segregation, like vertical and horizontal segregation, to further our understanding of the gender-equality paradox (Jarman et al., 2012). In this perspective, economic progress not only leads to women's empowerment and increased labor market participation but also to an increase in postmaterialist values and the opportunities for women and men to express their gendered selves, which strengthens horizontal gender segregation (Charles and Bradley, 2009; England, 2010; Charles, 2011). Thus, more recent research tried to explain the cross-county pattern of occupational gender segregation by pointing toward the strong persistence of gender-essentialist stereotypes and the increase of postmaterialist values, which both increase women's and men's opportunities to express their gendered selves (Charles and Bradley, 2009).

Against the backdrop of this academic discourse on the gender-equality paradox, the influences of two societal aspects seem to be at the core of the paradox regarding occupational expectations: the empowerment of women and post-material values. Both affect gender segregation in the labor market in different ways. Therefore, in the following, we will focus on these two aspects. However, both the old economic and neo-institutionalist perspectives and the new gender-essentialist approaches refer to processes taking place at the macro level for explaining changes in occupational gender segregation. Therefore, in the following, we discuss how these societal processes might affect adolescents' occupational preference formation at the individual level. Furthermore, we formulate several hypotheses derived from the theoretical explanation at the individual level, the opposing theoretical explanations of gender segregation at the macro level, and the gender-equality paradox.

### 3.1. Women's empowerment and gendered occupational expectations

In recent decades, women around the world have gained greater access to various societal domains, including education, the labor force, and political systems. In line with the neo-institutionalist perspective, women's empowerment at the macro level affects adolescents' occupational preference formation. At the individual level, this can be explained through the imitation of *same-sex role models*, which should matter mostly for girls' occupational expectations. The same-sex hypothesis assumes that children and teenagers tend to identify with the parent of the same sex (Eccles and Hoffman, 1984; Ruble et al., 2006). Daughters imitate the values, behaviors, and self-concepts of their mothers in order to learn how to develop into a competent female adult, while sons identify with their fathers (Bussey and Bandura, 1999). When developing occupational preferences, girls tend to orientate themselves toward the labor market status and occupations of their mothers, while the employment positions of fathers is more important for boys (Shu and Marini, 1998). Occupational expectations of children are thus less stereotypical if their same-sex parent works in a non-traditional occupation (Shu and Marini, 1998; Buchmann and Kriesi, 2012; Polavieja and Platt, 2014).

However, same-sex role models are not restricted to mothers and fathers but also include further relevant others, such as peers, teachers, or women and men in the media, meaning that girls and boys are able to abstract sex-appropriate behavior from their concrete observations of relevant others.^3^ Therefore, gendered occupational expectations not only reflect parental role models but also the actual employment and occupational distribution of men and women in the adult labor force (Xie and Shauman, 1997). For example, if girls observe a higher share of women working in management positions or in parliament, domains traditionally associated with male dominance, these positions are increasingly perceived as accessible for young women, too. In a similar manner, if girls observe more women working in formerly male-dominated occupations and professions, they associate these occupations as less traditional male and perceive them as appropriate occupational choices also for women (Xie and Shauman, 1997). Taking together the assumption of the neo-institutionalist perspective at the national level and the theory of same-sex role models at the individual level, we would expect that with rising levels of women's empowerment, girls are more likely to develop gender-atypical occupational expectations. However, it is not possible to derive such a clear assumption for boys, since they might continue to prefer stereotypically “male” professional and managerial occupations due to their high labor market rewards. In contrast, if girls and boys still observe a larger share of men and women working in gender-traditional occupations in their social environment, they are more likely to stick to gender-typical expectations.

**H1a**. In countries with increasing empowerment of women, girls will develop more gender-atypical occupational expectations.
**H1b**. In countries with decreasing occupational gender segregation, girls and boys will develop more gender-atypical occupational expectations.

### 3.2. Gendered beliefs, stereotypes, and self-expression values

However, most empirical research did not support the assumptions derived from evolutionary perspectives. Therefore, Charles and Bradley (2009) argue that modernization approaches cannot account satisfactorily for cross-national variations in occupational gender segregation since they underestimate the enduring cultural force of gender-essentialist ideologies, which are intensified by a strong Western cultural emphasis on values of individual self-expression and self-realization (see also Charles and Bradley, 2009; Charles et al., 2014). Gender beliefs and gender stereotypes are one of the most omnipresent social forces in societies and shape human behavior and various societal domains such as welfare states, labor markets, educational systems, and households. Thus, the central tenet of Charles and Bradley's is that the combination of gender-essentialist stereotypes and norms of self-expression reinforces the gender typing of curricular choice in modern societies (Charles and Bradley, 2009, p. 928).

During socialization in childhood and early adulthood, boys and girls develop values, skills, and self-concepts in accordance with these gender-typical normative expectations and values of their environment (Ruble et al., 2006), also regarding the gender-typing of occupations (Eccles, 1987). According to Xie and Shauman (1997), these occupational socialization processes not only depend on parents but are also conveyed to children through multiple sources in their social environment, including older siblings, teachers, peers, and the media. Therefore, social environment factors in general serve as socializing agents holding more or less stereotypical expectations regarding the gender-typing of occupations. These socializing agents are strongly influenced by a country's prevailing gender ideology, which represents the level of support for the division of wage work and caregiving work between men and women based on the notion of separate spheres (Davis and Greenstein, 2009).

Based on this internalization of normative expectations, self-expressive values, which are inherently linked to modernization processes, trigger a reproduction of gender essentialist beliefs. Young men and women use different cultural schemas to express their true “selves.” Due to increasing importance of self-expression values, increasing number of young people believe that they are responsible for meeting normative expectations according to gender essentialist beliefs and stereotypes (Charles and Bradley, 2009). In the course of modernization, value priorities have shifted from an emphasis on economic and physical security toward an increasing emphasis on subjective wellbeing, self-expression, and quality of life (Inglehart and Welzel, 2005). Even though the shift from survival to self-expressive values is accompanied by rising public support of gender equality (Inglehart, 2008), gender-essentialist beliefs nonetheless remain important for the development of human identity. Scholars believe that gender stereotypes evoke gendered occupational preferences by determining the evaluation of the self and others and by promoting standards of femininity and masculinity that must be met to avoid social disapproval (Correll, 2004; Charles, 2011). Therefore, self-expressive value systems tend to encourage the development and enactment of culturally masculine or feminine traits, including occupational expectations (Charles and Bradley, 2009). Furthermore, the increasing importance of self-expression in societies reproduces gender inequalities through actions based on cultural beliefs that define gender-appropriate behavior (Cech, 2013).

**H2**. In countries with increasing self-expressive values, boys and girls develop more gender-typical occupational expectations.

Many scholars conclude that gendered stereotypes and self-expression values might explain the persistence of occupational gender segregation even in the most developed societies (Correll, 2004; Charles and Bradley, 2009; England, 2010; Cotter et al., 2011) and thus the gender-equality paradox. However, only very little comparative research actually investigated how gendered stereotypes and self-expression values affect adolescents' occupational expectations, again with inconclusive results.

Although previous comparative research examined how different national characteristics shape occupational aspirations and expectations, the empirical findings are not consistent and inconclusive. Given the huge variation in observed outcomes and explanatory factors, methods, number of analyzed countries, and time points of observations, earlier results may not be generalized without further research. Moreover, most of these studies apply a cross-sectional design, which does not allow for more causal interpretations of their findings. Therefore, we formulated opposing hypotheses to reassess the assumptions behind the gender-equality paradox by addressing the impact of women's empowerment and self-expression values on young people's professional expectations.

In the following, we therefore examine how the gender-specific occupational expectations of two different youth cohorts within 26 European societies change from 2006 to 2018 and whether country-specific characteristics shape these changes in gender-specific occupational expectations. Against the backdrop of the state of comparative research on gender segregation and the gender-equality paradox, we first examine whether indicators related to women's empowerment follow the assumption of modernization theories or the gender-quality paradox. Second, we investigate whether gender stereotypes and self-expressive values, which both prevail in even the most modern societies, reproduce gender-typical occupational expectations.

## 4. Data, measures, and analytical methods

### 4.1. Data

To answer our overarching research questions and test our hypotheses empirically, we combined individual-level data with country-level information from various sources of two different points in time. We used PISA data from 2006 to 2018 for the individual level (OECD, 2009a, 2019), which we merged with country-level information from several sources, such as the Organization for Economic Cooperation and Development (OECD), the International Labor Organization (ILO), and the European and World Value Survey. Some of the national-level information is from earlier years, for instance the European and World Values Survey data.^4^ For our country sample, we selected 26 European countries to ensure a base level of homogeneity given that gender segregation varies widely between developed and developing countries (see e.g., Chang, 2004; Charles and Bradley, 2009). Due to missing information for some macro-level indicators, we could not include all EU-28 countries and had to exclude Croatia, Republic of Cyprus, Malta, and Romania.^5^

Although the PISA data set provides comparable measures across participating states in one year, it has been challenging to select appropriate variables to combine the PISA data with other information on the participating states for a longitudinal perspective. Thus, the selection of variables from the PISA data and the other sources was severely constrained by the limited availability of comparable measures across countries and over time. Thus, due to these data limitations, we could only include a small number of indicators in our following analyses, even though research has identified various institutional factors that affect young people's occupational expectations.^6^ Moreover, for some variables, we could only use data from other years. Despite these limitations, we were able to consider individual and country characteristics to analyze the change in gendered occupational expectations over time. In the following, we describe the dependent and independent variables in more detail at the different levels.

### 4.2. Dependent variable

We operationalize the occupational expectations of 15-year-old students in the selected countries using the PISA assessment question “What kind of job do you expect to have when you are about 30 years old?” This question is well-suited to capture realistic occupational aspirations (expectations), since it refers to occupational preferences that young adolescents consider realistic in terms of suitability and accessibility in adult life. To evaluate whether an occupational expectation is gendered, we calculated the share of women in each occupational category based on nationally representative labor force statistics from the ILO database for the years 2000/01. Afterwards, we merged the students' occupational expectations with the aggregated information about the share of women in the students' aspired occupations by using the 3-digit coding of the International Standard Classification of Occupations 1988. Due to the change in the ISCO coding between 2006 and 2018 (from ISCO-88 to ISCO-08), we decided to establish a reference point for coding the share of women in aspired occupations and defining the gender typicality of these aspired occupations. Therefore, we used information from the 2000/01 ILO data to define the gender typicality of aspired occupation for both years. This method has the following implications: the observed changes in gendered occupational expectations are not related to changes over time in the gender segregation of the labor market within countries. Thus, our results show the differences between the occupational expectations operationalized by the gender composition in occupations in 2000/01 and not the differences operationalized by the different gender composition in occupations in 2006 and 2018.^7^ Although we do not consider changes in the gender composition of specific occupations over time, we do consider changes in the general gender segregation in the labor market, as discussed later.

We considered two different types of dependent variables. First, we calculated the models with a metric outcome that includes the share of women in adolescents' occupational expectations. In addition, we also generated a categorical variable that differentiates between female-dominated (≥70% women), male-dominated ( ≤ 30% women), and gender-balanced (>30 and <70% women) occupational expectations. In our analyses, we used these categories to calculate three different models, that allow us to investigate whether effects are observed for either female-typical (vs. gender-balanced and male-typical) or male-typical (vs. gender-balanced and female-typical) or gender-balanced (vs. female-typical and male-typical) occupational expectations.

### 4.3. Independent variables

Our main independent variables are characteristics at the country level. Here we considered, on the one hand, information on the empowerment and participation of women in the labor market and, on the other hand, indicators of gender norms and self-expressive values. For the indicator of the first domain, women's empowerment, we included data on the female employment rate, the share of women in management, and the female-to-male ratio in parliament. Further, we considered information on the overall level of occupational gender segregation measured by the Index of Dissimilarity^8^ (DI). Most of the information was provided by the ILOSTAT (International Labour Organization, 2021) and OECD datasets and the Global Gender Gap Report (Hausmann et al., 2006, 2018; see also in the Supplementary Table A.1). For the second social domain—gender norms and self-expression values—we used the European and World Values Survey (Inglehart et al., 2020) to gain information on gender norms and the importance of self-expressive values. Attitudes toward working mothers, measured by the statement “A working mother can establish just as warm and secure a relationship with her children as a mother who does not work” in the European and World Values Survey, served as indicators of social gender norms. We estimated the mean per country, with higher values indicating more progressive gender norms. For the self-expression values, we used a modified version^9^ of the self-expression index provided by Inglehart and Welzel (2005). According to the authors, self-expression values give high priority to environmental protection, tolerance of diversity, rising demands for participation in decision-making in economic and political life, tolerance of outgroups (including foreigners, gays, and lesbians), gender equality, and a shift in child-rearing values (Inglehart and Welzel, 2005).

Since economic development interrelated with women's empowerment (e.g., Duflo, 2012) and has been considered as important factor to explain occupational expectations in other research (e.g., Charles, 2017; Stoet and Geary, 2022), we additionally included the Gross Domestic Product (GDP) per capita (World Bank, 2021) in our second-level models. Even though the impact of the economic growth of societies are not in the focus of our discussed factors above, it is an important aspect of the modernization theories, which is strongly related with women's empowerment and evaluation of values. Therefore, we will examine whether economic growth has an independent explanatory power.

To control for individual and social factors affecting occupational expectations, we further considered the following variables for each country. They are in line with previous literature from single-country studies. According to socialization theory, we included the Index of Economic, Social, and Cultural Status (ESCS) and the occupations of respondents' parents, as provided by PISA, because research shows that parents' gender roles model their children's occupational aspirations (Law and Schober, 2022). Furthermore, we considered migration status to account for the cultural role of parents and other significant people in the student community and the role of being in a migrant household for shaping occupational expectations (Akosah-Twumasi et al., 2018; Plenty and Jonsson, 2021). Finally, we control for age and competencies in math, literacy, and science measured by PISA. Educational research has analyzed the effect of educational performance on occupational aspirations (Wang et al., 2013). For instance, performance in math and reading was found to be linked to girls' educational aspirations, whereas only math was linked to boys' aspirations (Widlund et al., 2020). Another study found math achievement to be a “critical filter” to subsequent math careers (Shapka et al., 2006). At the individual level, a myriad of factors affect the development of occupational expectations; but school performance, migration background, and parental education play important roles in explaining occupational expectations (Valls et al., 2022). This information allows us to control the effect of individual variables at the first level (both gradual and categorical) so that we can calculate the effect of the country variables at the second level and disentangle them from the individual factors.

A detailed description of all the dependent and independent variables, including their coding and respective sources, is provided in the Supplementary Table A.1. An overview of the means over all independent country variables shows that there is variation between the countries and over time (see Supplementary Table A.2).

### 4.4. Sample selection

The total 2006 PISA sample includes over 398,750 students from 57 countries; the 2018 data includes 617,259 students from 79 countries. Given the high variance of gender segregation between developed and developing countries, we chose to focus only on developed countries. Further, we focus on European countries to ensure that, to a certain degree, these states share common political norms and goals. In addition, we excluded countries for which information on any of the independent variables at the individual or state level was not available. For the individual data of PISA, we included only cases that had valid values for all variables of interest presented in both survey years. This truncation left us with 125,485 cases for 2006 and 139,075 cases for 2018 on the first level and 52 country cases (26 countries for each time point) in the European region on the second level.^10^

### 4.5. Analysis strategy

To answer our first research question—whether the occupational expectations of girls and boys changed over time and countries—we looked at the differences in gendered occupational expectations between the 2006 and 2018 student cohorts by gender. Initially, we calculated the mean differences for the share of women in the aspired occupation by gender and time (see Supplementary Table A.3). Using the mean, we could only see changes at the level of gender-atypical or gender-typical choice but not changes in the variance between the ends of the distribution. Therefore, we additionally compared the distribution for three different categories (gender-atypical, gender-balanced, and gender-typical) between 2006 and 2018 (see Supplementary Table A.4). For the final illustration of the change in young people's gendered occupational expectations between 2006 and 2018, we calculated the change in percentage of gender-typical and gender-atypical occupational expectations for girls and boys separately by controlling for compositional effects^11^ and plotted the values. The pattern of these plots shows whether changes were more between gender-typical and gender-atypical expectations or more toward gender-balanced expectations.

Regarding our second research question—whether country-specific characteristics shape the changes in occupational expectations—and to test our hypotheses, we had to consider two particularities when choosing the analysis strategy for our main analysis. First, our hypotheses especially address the changes over time in the dependent and independent variables. Hence, we used a longitudinal analysis strategy by applying a fixed-effects panel regression^12^ that allowed for stronger causal inferences about potentially influencing state characteristics. In comparison to cross-sectional analysis, the longitudinal approach enabled us to control for unobserved heterogeneity in time-invariant variables between the countries. The disadvantage of this method is that we cannot make any statements about the influence of time-invariant factors. Second, in the PISA sampling procedure, students are nested in schools and countries. Thus, the data can be represented as a hierarchical structure with three levels (students, schools, and countries). To consider this data structure, we used a multilevel method. Because of our focus on country-level factors explaining gendered occupational expectations and because the number of observations at the country level limited our methodological choice, we only consider the individual level of the students and the country level in our analytical strategy.

Specifically, we used a two-step approach for our longitudinal multivariate analyses to study the multilevel data. The two-step approach takes account of the fact that individual-level effects might vary across countries. By means of this approach, any further distributional assumptions were imposed (Gebel and Giesecke, 2011). In the first step, we pooled all individual data and included the country-variable as a dummy variable.^13^ We calculated four linear regression models (by gender and year) for each dependent variable: the share of women in the aspired occupation, the female-typical, the male-typical, and the gender-balanced occupational expectations (see Supplementary Tables A.6–A.9). To account for the sampling variance in PISA, we applied state weights in the calculation (OECD, 2009b). Further, we calculated the plausible values of the competency tests using the stata modules^14^ provided for the PISA data. In the second step, we used the resulting country coefficients as dependent variables for the state-level regression models. To acknowledge that the dependent variable consists of estimated values from the first step and hence introduces biased standard errors in the second step, we applied the feasible generalized least squares (FGLS) estimator as suggested by Lewis and Linzer (2005). With this approach, we calculated panel-regression^15^ models with fixed effects to determine which changes in country-level variables were associated with changes in the dependent variables called gendered occupational expectations.

As the pattern of gender-quality paradox presented by former research was based on cross-sectional data, we additionally calculated models with pooled data (cross-sectional) from 2006 and 2018 to illustrate the difference in results generated using the different methods. By comparing these different methods, we test whether the gender-equality paradox also occurs in our sample when we use the same approach as previous studies and test whether our methodological choice to use longitudinal approaches is justified.

Given the small number of clusters (countries), the type-I error rate for the significance tests of the coefficients is increased (McNeish and Stapleton, 2016). Moreover, regression models with a small number of clusters might not be robust regarding sample composition and the chosen variable. To test if the results vary between different sample compositions, we ran different models excluding each country once and compared the results.

## 5. Results

In the following, we answer our research questions about the changes over time and test our hypotheses on the effect of different societal factors on the evolution of gendered occupational expectations. Further, against the background of the gender-quality paradox discussed above, we test our hypotheses. For that purpose, we will describe the changes in occupational expectations from 2006 to 2018 by country and gender and present the results of the panel regression within a multi-level approach.

### 5.1. Gendered occupational expectations over time

**Figure 1:**
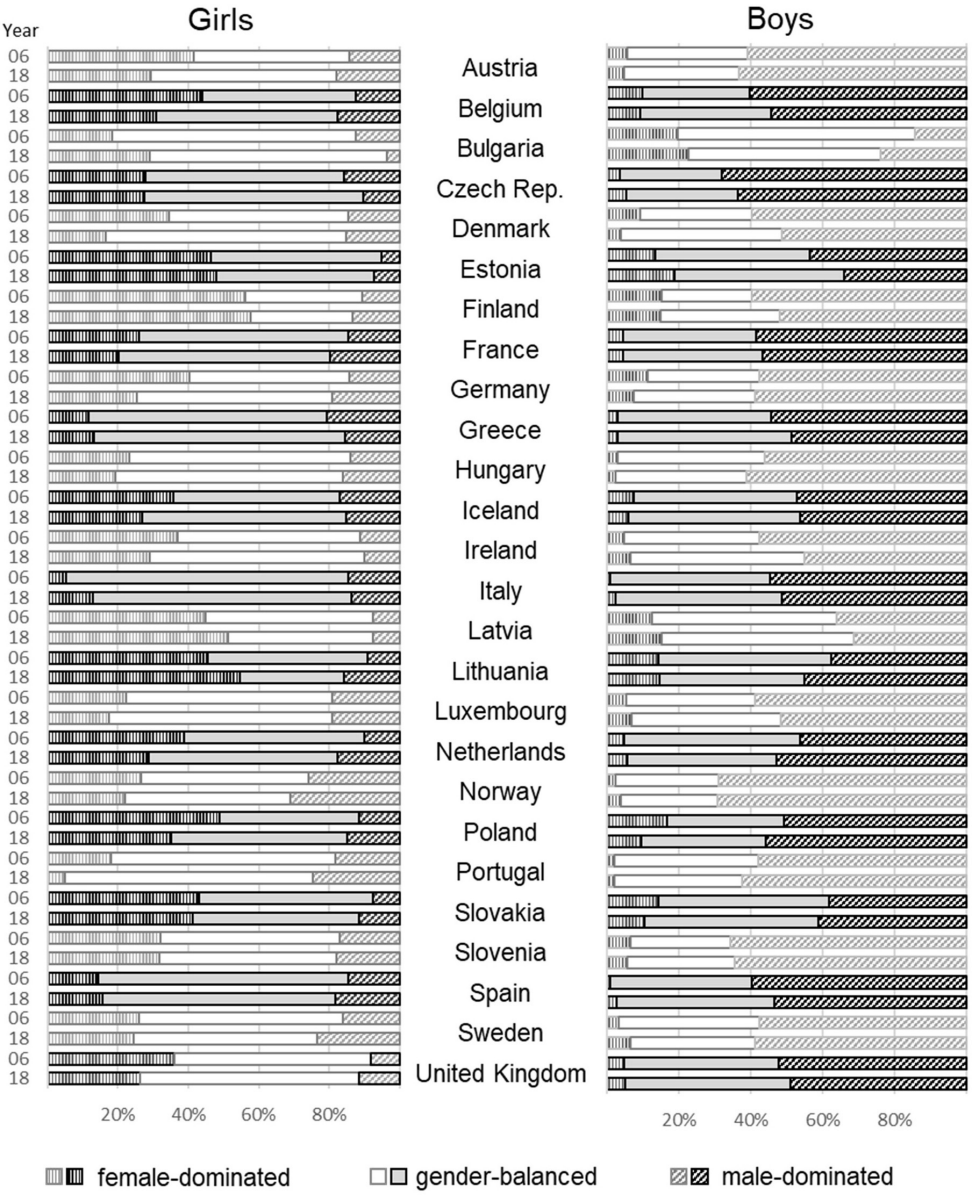
Distribution of students' occupational expectations by gender and year.

At first glance, Figure 1 shows the distribution of students with female-dominated, gender-balanced, and male-dominated expectations by gender, country, and year.^16^ The overall pattern shows that in general, girls aspire more to female-dominated occupations (bars with vertical lines) and boys aspire more to male-dominated occupations (bars with diagonal lines). Even though the pattern seems to be similar across countries, the number of young people with gender-balanced occupational expectations (white/gray bars) varies across countries, especially for girls. For example, in Bulgaria, most young girls aspire to gender-balanced occupations, whereas in Finland, most girls aspire to female-dominated occupations. Similar patterns emerge for boys in these two countries. Together with the other countries, our results are in line with the above-discussed gender-equality paradox. The countries more progressive in terms of gender equality show descriptively stronger gender segregation in occupational expectations.

**Figure 2:**
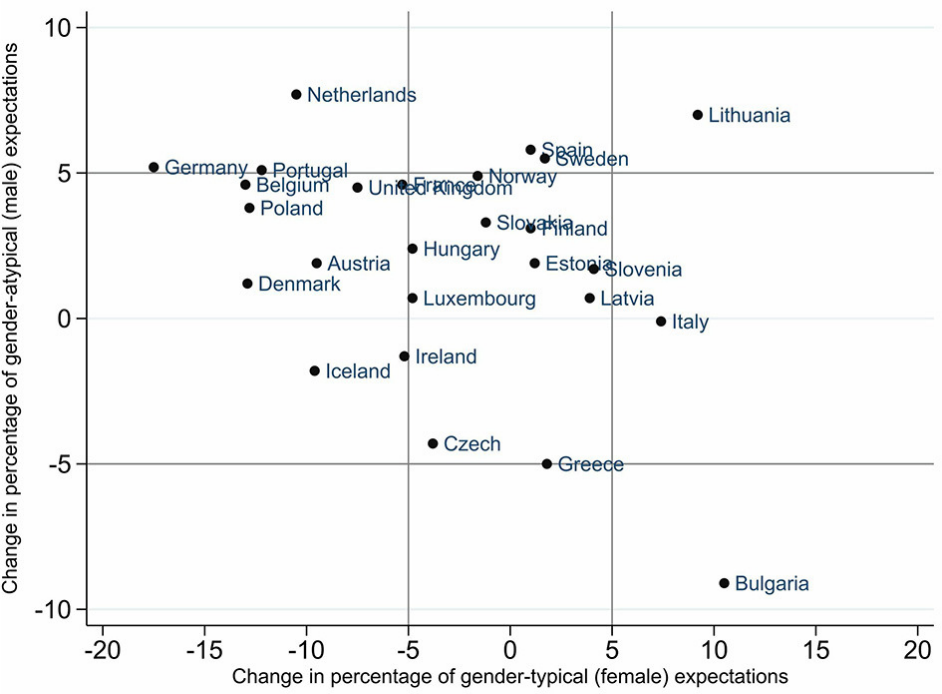
Change in percentage points of gender-(a)typical occupational expectations of girls from 2006 to 2018. Gender-typical expectations: occupation with more than 70% persons of the same sex. Gender-atypical expectations: occupation with fewer than 30% persons of the same sex. Cut-off at 5% (gray lines).

**Figure 3:**
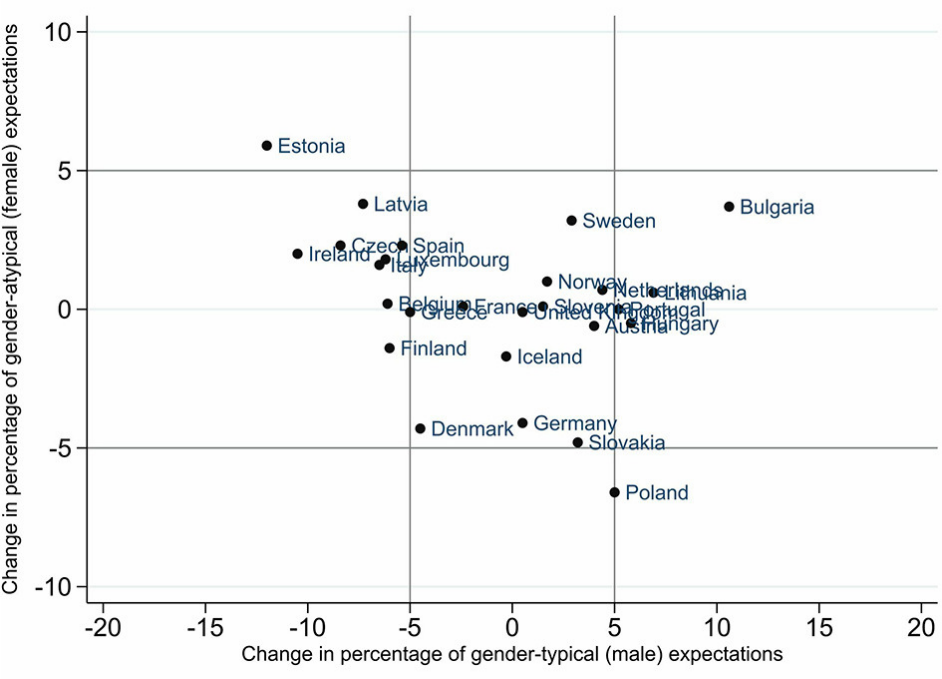
Change in percentage points of gender-(a)typical occupational expectations of boys from 2006 to 2018. Gender-typical expectations: occupation with more than 70% persons of the same sex. Gender-atypical expectations: occupation with fewer than 30% persons of the same sex. Cut-off at 5% (gray lines).

To answer the question of how gendered occupational expectations changed over time, Figures 2, 3 show the change in the percentage of gender-typical and gender-atypical occupational expectations of girls and boys from 2006 to 2018. With the help of these illustrations, the different countries can be divided according to whether occupational expectations have moved more toward gender-typical or gender-atypical expectations or whether they tend to converge in a gender-balanced way. In both figures, the cut-off at 5 percentage points is marked with gray lines, meaning the whole area is divided into nine sections. As a result, very different types of changes emerge.

For example, Figure 2 shows that Lithuania saw growth both in the group of girls with gender-typical (+9 p.p.) and gender-atypical (by +7 p.p.) occupational expectations. This means that girls in Lithuania aspired more to occupations with a larger share of females or males (above 70% and below 30%) and less to gender-balanced occupations in 2018 than in 2006. In Bulgaria, by comparison, the group of girls with gender-typical occupational expectations (female-dominated occupations) grew by over 10 percentage points, whereby the group of girls in Bulgaria aspiring to gender-atypical occupational expectations (male-dominated occupations) declined by about 8 percentage points.

Regarding boys' occupational expectations, we observed much fewer changes from 2006 to 2018. In many more countries, the changes for boys are about 5 percentage points in both groups: gender-typical and gender-atypical expectations. Further, the changes to be observed were on the horizontal axis, that is, changes toward gender-typical expectations. For example, boys in Bulgaria chose more male-dominated occupations (by +10 p.p.). In contrast, boys in Estonia aspired to less male-dominated occupations (−12 p.p.).

The graphs show some interesting patterns of development for both genders for each country, leading to assumptions about a potential change in gender segregation in the labor market. In the Bulgarian case, where girls and boys change their expectations toward more gender-typical occupations, we would thus expect a more gender-segregated labor market in the future. In the case of Denmark, where girls aspired less to female-dominated occupations and boys aspired less to female- and male-dominated occupations, meaning that both groups showed increases in the group of gender-balanced occupational expectations, we would expect a decrease in future labor-market gender segregation. To check whether this trend holds for all European countries in the sample, we additionally calculated the change in occupational expectations as a metric variable and for all three categories as a nominal variable. The results in Supplementary Table A.10 confirm that, on average, young people's occupational expectations have become more gender-balanced between 2006 and 2018.

Overall, the illustration of changes in gender-specific occupational expectations shows that career expectations differed between the 2006 and 2018 student cohorts. These differences were only evident through the analysis of distributional differences and not through the analysis of mean differences, which did not reveal variation in the pattern of change. Magnitude and direction vary across countries and genders, so patterns of change can be distinguished in terms of increasing, decreasing, or stable gender segregation in occupational expectations and in terms of potential trends in gender segregation of the labor market.

### 5.2. Institutional effect on change in occupational expectations

As shown in the previous section, gender-specific expectations changed over time. The following section analyzes the institutional time-varying factors that explain these changes and whether our hypotheses can be verified. Table 1 presents the results of the multi-level panel regression model based on a two-step approach and, additionally, the results of a regression model with pooled data. To identify the changing country factors that explain the changes in occupational expectations, we included all items of the different societal spheres—women's empowerment, labor market structure, gender norms and values, and economic wealth—in the models simultaneously.

**Table 1:** Results of the regression models of the share of females in aspired occupation by gender.

	**Panel regression with fixed effects**		**Regression with pooled data**	
	**Girls**	**Boys**	**Girls**	**Boys**
**Women's empowerment**				
Female employment ratio	**−0.018** ^***^	**0.011** ^**^	**0.004** ^*^	0.000
Women in parliament	**−0.311** ^**^	**0.184** ^*^	**−0.106** ^*^	0.009
Women in management	−0.448	0.014	−0.005	−0.196
Dissimilarity index	0.000	−0.001	**0.009** ^***^	−0.003
**Cultural norms and values**				
Gender norms	−0.231	0.081	**0.180** ^*^	0.233
Self-expression	**−0.085** ^***^	**0.046** ^**^	−0.003	**−0.042** ^**^
**Economic wealth**				
GDP	−0.004	0.003	0.000	**−0.002** ^*^
Year (Ref. 2006)			−0.018^***^	0.012^***^
Constant	2.405^***^	−0.643^**^	36.078^***^	−22.823^***^
Observations	52	52	52	52
$\hat{\sigma }$	0.036	0.027	0.063	0.090
Average ω	0.007	0.007	0.007	0.007

Significant coefficients of the independent variables are in bold.

*** p < 0.01.

** p < 0.05.

* p < 0.1.

The results of the panel regression of the metric outcome variable (share of females in aspired occupation) reveal that the variables of women's participation and the self-expression values show a significant effect for both girls and boys. Remarkably, the directions of the effects are not in line with the gender-equality paradox. If women's participation and self-expression values are higher, girls tend to aspire to occupations with fewer females and boys tend to aspire to occupations with more females. These results are not supporting the gender-equality paradox pattern and our hypotheses H2. Thus, the association between the level of self-expression values and the share of females in the aspired occupation is contrary to the above assumption (H2) that a higher level of self-expression values would lead to a higher level of segregation. Our results are in line with our first hypothesis (H1a) and the theoretical assumption that an increase in women's empowerment contributes to a desegregation of occupational expectations. This is not only true for girls but also applies to boys. Further, changes in the horizontal gender segregation in the labor market does not affect the occupational expectations of boys and girls (H1b).

As previous research predominantly used cross-sectional data, we want to compare the result of our longitudinal approach with the results of a cross-sectional approach. A comparison between the results of the panel regression with fixed effects (first two models) and of the linear regression model with pooled data (third and fourth model) shows that conclusions about the effective factors differ between the methods. For example, in the panel model of the girls (first model), the dissimilarity index and the gender norms are not significant, whereas these two items are significant in the pooled model (third model). The most pronounced difference between the models can be found regarding the direction of the effect for some variables. In the panel fixed-effects model of the boys (second model), the association between self-expression and the share of females in the aspired occupation point in the opposite directions, as in the pooled model (fourth model). For the models of the girls, the same pattern can be seen for the female's employment rate. These different results are based on the fact that both methods analyze different types of variance: the fixed effect model used within-country variance, the pooled model (cross-sectional) used between-country variance. Therefore, when the between-variance of the country characteristics overrule the within-variance in the pooled models, the direction of the coefficient changes. Controlling for unobserved heterogeneity and only considering within-variance in the panel regression with fixed effects reveals a different picture that leads to a totally different conclusion about the association between gender norms and values and the gender segregation of occupational expectations. The results of the pooled models are in line with the gender-equality paradox hypothesis. However, the results of the fixed effects models refute it.

As shown in Section 4.1, the pattern of changes in occupational expectations emerges more clearly when separating occupational expectations into three different categories. Furthermore, we observed different patterns in changes across countries. Therefore, we additionally calculated panel regression models by gender for three different outcomes: female-dominated occupation, male-dominated occupation, and gender-balanced expectations vs. the others (see Table 2).

**Table 2:** Results of the regression models of the categorial outcomes by gender.

	**Female-dominated**		**Male-dominated**		**Gender-balanced**	
	**Girls**	**Boys**	**Girls**	**Boys**	**Girls**	**Boys**
**Women's empowerment**						
Employment	**−0.038** ^***^	−0.001	**0.007** ^***^	**−0.015** ^*^	**0.031** ^**^	**0.016** ^*^
Parliament	**−0.647** ^**^	−0.048	0.048	**−0.385** ^**^	**0.600** ^**^	**0.432** ^**^
Management	−1.149	−0.167	−0.063	−0.102	1.231	0.266
Dissimilarity index	−0.004	−0.002	−0.003	0.004	0.008	−0.002
**Cultural norms and values**						
Gender norms	−0.540	0.009	0.035	0.027	0.502	−0.036
Self-expression	**−0.188** ^***^	**−0.021** ^**^	**0.047** ^***^	**−0.067** ^**^	**0.140** ^**^	**0.088** ^**^
**Economic wealth**						
GDP	−0.011	−0.001	0.001	−0.005	0.010	0.006^*^
Constant	4.686^***^	0.442^***^	−0.354^**^	1.722^***^	−3.338^***^	−1.164^**^
Observations	52	52	52	52	52	52
$\hat{\sigma }$	0.085	0.013	0.013	0.043	0.080	0.048
Average ω	0.014	0.008	0.012	0.016	0.016	0.016

Significant coefficients of the independent variables are in bold.

*** p < 0.01.

** p < 0.05.

* p < 0.1.

Overall, the results show a pattern of the coefficient similar to the former model of the metric outcome (share of women in occupation). For all models, the self-expression values have a significant impact on the occupational expectations of boys and girls. Likewise, the coefficients of women's participation in the labor market and in parliament show a significant effect in almost all models. It is noticeable that the expectation of boys to work in a female-dominated occupation is least influenced by the country factors. This finding shows the stronger stability of boys' avoidance of female-dominated occupations regarding changes of institutional characteristics. For boys, institutional factors seem more likely to cause a change between the expectations of male- and gender-balanced occupations.

Our separate analyses along the three different categories show that the significant effects of the country factors lead young people from gender-typical expectations to gender-balanced occupational expectations. Thus, women's higher participation in the labor force and in parliament, and more pronounced self-expression values decrease girls' expectations to work in a female-dominated occupation, whereas the same factors decrease boys' expectations to work in a male-dominated occupation. The positive associations in both models of gender-balanced expectations indicate that the institutional factors with a significant effect influence the occupational expectations of young people toward gender-balanced occupations. Comparing the results of girls and boys shows that changes in country characteristics lead to more gender-balanced and even male-dominated occupational expectations for girls but only toward gender-balanced occupational expectations for boys (see Supplementary Table A.10).

To test whether our results are robust to sample composition, we repeated all longitudinal analyses and excluded one country from the analysis at a time. A cumulation of the results of each of the 26 different models reveals that our results are stable, especially for women's empowerment and self-expression values (Supplementary Table A.11). Further, we tested whether the consider factors are highly correlated. We found modest to high correlations, mostly between women's empowerment and self-expression values, as implied by the theoretical considerations (see Supplementary Tables A.12, A.13). Additionally, we calculated models with only one independent variable on the second level to see whether the inter-item-correlation affected the results in the overall model (see Supplementary Table A.14). Even though the single-item models showed that almost all independent variables had a significant effect, in the multiple models only the net effects of the theoretically most relevant measures remained significant. Thus, these effects were mediated through the other independent variables in the overall models.

Overall, based on our results, we could not verify the assumption about the role of women's empowerment and self-expression values regarding the gender-equality paradox. Even though we observed the same counterintuitive descriptive pattern—more pronounced gender segregation of occupational expectations in more gender-progressive countries than in other countries—we did not find a positive association between this segregation and more progressive country characteristics. On the contrary, using a longitudinal approach that considers only within-country changes, we demonstrated that an increase in women's participation and self-expression values leads to desegregation. Furthermore, our separate analyses for girls and boys indicated that girls are more influenced by the observed country characteristics than boys. This pattern is in line with the policy focus on desegregating the labor market by encouraging girls to pursue STEM subjects. Moreover, this observation also makes theoretical sense because the theoretical considerations and the measured country variables are predominantly oriented toward how girls' occupational aspirations are influenced.

## 6. Summary and discussion

Recent decades have seen a very significant increase in women's participation in the labor market almost worldwide. However, horizontal gender segregation in the labor market persists and has become even stronger in more gender-progressive countries. One of the main explanatory factors for this counterintuitive pattern is the persistently gendered nature of occupational expectations, which results in the gender segregation of labor. Drawing on the comparative research on the gender-equality paradox, we investigated how the gender-specific occupational expectations of different youth cohorts in 26 European societies change over time and whether country-specific characteristics influence these changes in gender-specific occupational expectations.

Our first descriptive results on the distribution of occupational expectations across European countries are in line with the gender-equality paradox pattern. Thus, young people in countries known as more progressive regarding gender equality had more gender-segregated occupational expectations. Nevertheless, a comparison between different student cohorts' occupational expectations showed that, on average, young people's occupational expectations became more gender-balanced between 2006 and 2018. Further, a cross-country comparison of the changes over time revealed that the patterns of change varied between the European countries. Here, magnitude and direction differed between countries and genders, so that patterns of change can be distinguished in terms of increasing, decreasing, or stable gender segregation in occupational expectations.

Notwithstanding the limitations of our study, which are outlined in the next section, the results of our longitudinal analyses within a multilevel approach revealed that, on the one hand, women's participation in the labor market, their participation in parliament, and general societal self-expression values indeed influence occupational expectations. On the other hand, these country factors contributed to a gender desegregation of occupational expectations within countries. Thus, in countries with an increase in women's empowerment and self-expression values, girls' expectations of working in a female-dominated occupation decreased and boys' expectations of working in a male-dominated occupation decreased. For both genders, these changing country characteristics changed the occupational expectations more toward gender-balanced occupational expectations. Earlier research that focused only on gender-typical or gender-atypical occupational expectations failed to capture this remarkable pattern. Even though we initially observed the same counterintuitive pattern—namely that gender segregation in occupational expectations is more pronounced in more gender-progressive countries—our finding on changes *within* countries did not verify previous assumptions about the mechanisms underlying the gender-equality paradox.

In contrast to the counterintuitive pattern of the gender-equality paradox mostly resulting from cross-sectional studies, our focus on changes within countries indicates that growth in aspects associated with societal modernization, such as women's empowerment and increased self-expression values, lead to a desegregation of young people's occupational aspirations. Thus, our results are more in line with the assumptions of modernization theories mentioned above. Since in modern societies, women's empowerment is higher and the prevailing gender ideology is generally more egalitarian, gendered occupational expectations decline, too. Researchers examining the gender-equality paradox in other areas already demonstrated that changing from a cross-sectional to longitudinal research design can profoundly alter results and corresponding conclusions. For instance, Fors Connolly et al. (2020) showed that gender differences in personality traits are only linked to gender equality in a cross-national, but not in a longitudinal perspective within countries.

Since our study focused on how institutional and cultural changes within a country affect adolescents' occupational expectations, we could not provide additional explanations for the counterintuitive differences between countries. Nevertheless, research on gender segregation in the labor market might already provide some explanations for differences between cross-sectional and longitudinal designs. One possible point of departure concerns the historical development of the welfare state, which varies considerably across the observed countries. For example, Mandel and Semyonov (2006) showed that especially in the Nordic countries, the welfare state expanded substantially in earlier decades, thereby increasing women's employment opportunities in the public sector in particular. This might explain why cross-sectionally, occupational gender segregation is still most pronounced in the Nordic welfare states. However, our results additionally demonstrate that also in these countries, increasing women's empowerment and stronger self-expression values contribute to a desegregation of occupational expectations over time.

Comparative research on young people's occupational expectations has been challenging to conduct given the lack of comparable data between countries and over time. In this respect, our study also suffers from some limitations in terms of data and sample size. First, although we could include almost all European countries in our analytical sample, the results based on a sample of 26 countries might be less robust in terms of sample composition. Second, due to the very limited data availability we could only use data of two time points and a very coarse measurements of institutional and cultural factors to investigate gendered occupational aspirations. Thus, it would have been desirable—but not feasible due to the limited data availability for the included countries—to use more time points and more detailed measurements of gender ideologies, as discussed by several researchers (e.g., Knight and Brinton, 2017; Grunow et al., 2018), and to consider additional state characteristics, such as the size of the welfare state or changes in salaries. Because of the coarse measurements, we cannot rule out the possibility that other unmeasured time-varying country characteristics influenced young people's expectations. Moreover, conclusions about the evolution of gender segregation in the labor market based on the study of young people's occupational expectations should be drawn with caution. Even though research has shown that occupational expectations translate into gender-specific educational choices and gender-specific occupational placements, the actual outcomes may differ significantly from the original individual expectations. This is because selection processes in the labor market, for instance through competition or discriminatory hiring practices, have not been studied. A comparison between gender segregation of occupational expectations and actual labor market segregation has shown that young people's expectations are less segregated than the labor market (Hillmert, 2015). Furthermore, the high number of missing values for the occupational expectations item implies that a large proportion of young people had not yet thought about or decided on their career plans at this early stage and were excluded from the study. These missing values vary between genders and are also very different from country to country and should be considered when interpreting the results.

In light of our methodological limitations, we would like to highlight three aspects for further research. First, given that the number of adolescents who could not answer the questions on their occupational expectations in PISA varies across countries, additional research on the career expectations and final decision of adolescents in this specific group is very important. Second, it would be fruitful to also compare longitudinal data at the individual level across countries, as the development of gender-specific occupational expectations at the individual level might also be influenced by national characteristics. Third, due to the limited scope of our study, further research with a much broader framework is need to understand the complexity of the interplay of individual and institutional factors that shape occupational expectations and gender segregation on the labor market (for an overview, see Anker, 1998; Charles, 2011). A wide range of research on the gender segregation in the labor market has identified many more national characteristics that affect segregation on the labor market, like the educational system (e.g., Smyth and Steinmetz, 2015), the size of the welfare state (e.g., Mandel and Semyonov, 2006), policy measures (e.g., Bettio, 2002), job and labor shortages in specific fields, and technological change (for a discussion, see Rubery, 2019). Although these factors only indirectly shape occupational expectations by influencing the structure of the labor market in terms of gender segregation, it is essential to look at them in order to get a full picture of the social mechanisms that influence young people's occupational expectations. With our focus on country characteristics that are especially addressed by the gender-equality paradox, we filled a specific gap in the research. Nevertheless, further research with a broader perspective is highly recommended. This research should include the labor supply-side and labor demand-side approach drawn, for instance, from human capital theory (e.g., Polachek, 1981; see also criticism by England, 1982; Anker, 2001).

From a policy perspective, the gender-equality paradox might be less problematic, because horizontal segregation comes with less vertical gender segregation. Especially vertical gender segregation in the labor market is associated with gender differences in power and income. Nevertheless, a growing body of research highlights the benefits of a diverse labor force and concludes that horizontal labor market segregation is an obstacle to societies in several respects. For example, research on workforce diversity has shown that group productivity is particularly high in diverse groups, or that the participation of women in boards contributes to higher returns in firms (van Knippenberg et al., 2004; Ali et al., 2011; Post and Byron, 2015). This is because high group diversity leads to cognitive diversity, for example, due to differences in experience, expertise, attitudes to risk and collaboration, but also sociocultural backgrounds.

## Data availability statement

Publicly available datasets were analyzed in this study. This data can be found at: https://www.oecd.org/pisa/data/; https://data.worldbank.org/; and https://ilostat.ilo.org/data/. For the three-digit coding of the occupations we used ILO data from http://laborsta.ilo.org/STP/guest (accessed 2013) that is not longer available.

## Ethics statement

Ethical review and approval was not required for the study on human participants in accordance with the local legislation and institutional requirements. Written informed consent to participate in this study was provided by the participants' legal guardian/next of kin.

## Author contributions

ME, KL, MH, and AM contributed to the conception and design of the study. ME and AM were in charge of the data collection. ME organized the database, performed the statistical analysis, and wrote the first draft of the manuscript. KL and AM revised the manuscript. All authors contributed to reviewing the manuscript critically for important intellectual content, reading, and approving the submitted version.

## Footnotes

^1^Although cross-national comparative studies mostly focus on institutional factors that affect young people's career expectations, there is a broad body of research on gender differences at the individual level that we do not discuss in detail here. For example, research has pointed to the importance of the following factors in the individual level for understanding gendered occupational expectations and choices: gender differences in math, science, or reading performance (Helbig and Leuze, 2012; Riegle-Crumb et al., 2012), self-esteem (Magnusson and Nermo, 2018), course-taking patterns in upper secondary school (e.g., Lörz and Schindler, 2011; Mann and DiPrete, 2013), life and career plans (e.g., Morgan et al., 2013; Weeden et al., 2020), and social influences through parents (e.g., Polavieja and Platt, 2014; Busch-Heizmann, 2015; Gabay-Egozi et al., 2015), peers (e.g., van der Vleuten et al., 2018; Raabe and Wölfer, 2019), and the school environment (e.g., Legewie and DiPrete, 2014). Nevertheless, we consider important individual-level factors in our analytical strategy and models.

^2^Furthermore, the distinction between STEM and non-STEM occupational aspirations is not equivalent to a distinction between gender-atypical and gender-typical occupational aspirations, because the share of female varies between field with in STEM fields. For instance, biological and agricultural science are female-dominated fields within the STEM-fields (see Mann and DiPrete, 2013).

^3^See Legewie and DiPrete (2014) for a similar argument on how the gender segregation of extracurricular activities in high schools might affect the development of STEM aspirations.

^4^This concerns mainly our indicator on cultural gender norms. However, we do not think this is a problem because gender norms begin to affect occupational aspirations in childhood. At the same time, gender norms are changing only very slowly, which is why a lag of some years seems acceptable.

^5^Even though Iceland, Norway, and Great Britain are not members of the European Union, we included Iceland and Norway in our sample because both are strongly integrated into the European Union through the Agreement on the European Economic Area and the Schengen Agreement. Great Britain left the EU after the data used were collected.

^6^For example, not all countries provided relevant information on the education system (e.g., France had no information on the gender composition of schools) and not all countries provide information on the share of labor in the public and private sector (e.g., Italy, Czech Republic, and Iceland).

^7^In the case where we would be able to consider changes in the gender composition of occupations in the definition, we may not see a change in occupational expectations in specific occupations, although there have been changes. For example, if girls were more likely to aspire to medical professions in 2006 and then more likely to aspire to legal professions in 2018, where the proportion of women has increased over the past decade, we would not see a change because girls preferred a female-dominated profession in both years. Thus, we would find no change over time, although young girls' expectations have changed over time. Therefore, our approach is consistent with the methodologies of studies that examine expectations for specific occupations, such as careers in STEM fields.

^8^This measure of gender segregation in the labor market is based on the work by Duncan and Duncan (1955). It gives the proportion of women (or men) who would need to change their occupation to achieve an occupational gender composition that equals the gender composition of the labor market.

^9^Because not all items of the original version were used in the later values survey, we created a new index based on the left original items and the results of a reliability analyses. Based on this, the items “Post-Materialist” index (4-item), “Justifiable: Homosexuality,” and “Justifiable: Abortion” were used for the adapted index for self-expression.

^10^The share of missing values in the dependent variable on the individual level varies across countries and gender. To handle these missing values, we follow other studies and exclude students with missing expectations rather than impute their values, as, for example McDaniel (2016), Blasko et al. (2018), and Hägglund and Leuze (2021) have done.

^11^The values are based on linear regression models with individual weights provided by PISA and considering clustered standard errors for the schools (see Supplementary Table A.5). In these models, age, ESCS, migration background and gender type of father's and mother's occupation are included as control variables.

^12^Since we consider only two time points the results of fix-effects models are a first-difference estimator.

^13^By using the prefix *ibn*. in stata for the country variable, we kept all cell means for the country in the regression models. Thus, the results provided for all countries include a coefficient that we could use for the second step.

^14^The command “pv:” compute estimates plausible values for each test domain.

^15^To account for the different countries' weight, which varied over time, we used the areg command instead of the xtreg command in stata for the calculations.

^16^As discussed in Section 4.2 the definition of the gender typicality of the aspired occupations of both years refer to the gender compositions in occupations from 2000/01.
