# Supplementary material for: Do women's empowerment and self-expression values change adolescents' gendered occupational expectations? Longitudinal evidence against the gender-equality paradox from 26 European countries
Source: Front Sociol. 2023 Jun 9;8:1175651. doi: 10.3389/fsoc.2023.1175651 (PMC10288873; doi:10.3389/fsoc.2023.1175651)
Supplement: Supplementary file 1 [file Table_1.docx]

Supplementary Material

**Do Women’s Empowerment and Self-Expression Values Change Adolescents’ Gendered Occupational Expectations?**Longitudinal evidence against the gender-equality paradox from 26 European Countries

Melinda Erdmann^*^, Agustina Marques Hill, Marcel Helbig, Kathrin Leuze

* Corresponding author: melinda.erdmann@wzb.eu

# Supplementary Figures and Tables

| **Table A.1:** Overview of measures and data sources | |
| --- | --- |
| **Variable** (Source parentheses) | **Definitions and details** |
| **Dependent variable** |  |
| Gender-typing of occupational expectation (metric) (PISA 2006, PISA 2018, ILO Segregate Database) | Share of women in each occupational category (ISCO 88 3-digit) in every country aspired by 15-year-old girls and boys at the age of 30 based on PISA 2006 and 2018 data merged with data from nationally representative labor force statistics mainly for the year 2000/2001. |
| Gender-typing of occupational aspiration (categorical)  (PISA 2006, PISA 2018) | “female-dominated occupation” (share of women in occupation 70 percent and above) “gender-balanced occupation” (share of women in occupation more than 30 and less than 70 percent) “male-dominated occupation” (share of women in occupation 30 percent and below) |
|  |  |
| **Independent variables** |  |
| ***Country-level variables*** |  |
| Female to male employment rate (ILO Segregate Database) | Women’s employment rate in 2006 and 2018. |
| Women in Parliament (Global Gender Gap Report 2006 & 2018) | Female-male ratio in national parliaments (females/males) |
| Women in Management  (ILO Segregate Database) | Female rate in management positions |
| Dissimilarity Index (ILO Segregate Database) | Proportion of women (or men) who would need to change their occupation in order to achieve an occupational sex composition that equals the sex composition of the labor market. $D=\sum_{j=1}^{J} \vert(F_{j}\left\vert F \right)-(M_{j}\vert M)\vert x 100 x \frac{1}{2}$Fj = N women in occupation j F = N women in the labor force Mj = N men in occupation j M = N men in the labor force |
| Gender norms  (European and World values survey 1999 & 2008, Norway 1996 & 2008) | Indicator of attitudes towards the gender roles based dichotomized country mean of the following item: “A working mother can establish just as warm and secure a relationship with her children as a mother who does not work” |
| Survival/self- expression values ([World Value Survey 1999 & 2017](https://europeanvaluesstudy.eu/methodology-data-documentation/data-and-documentation/)) | The self-expression index is based on Inglehart and Welzel (2005). We use a short index including the items of “Y002: Post-Matrialist” (Index with items), “F118: Justifiable: Homosexuality” and “F120: Justifiable: Abortion” |
| Gross Domestic Product (GDP) (World Bank) | Gross domestic product per capita in 2006 and 2018, current prices in US dollars |
| ***Individual-level variables*** |  |
| Socioeconomic status  (PISA 2006, PISA 2018)^1^ | Index of economic, social, and cultural status (ESCS), as provided by PISA |
| Migration status  (PISA 2006, PISA 2018) ^1^ | Dummy variable 0 “native” 1 “first- and second-generation migrant” |
| Mathematical competencies, Science competencies, Reading competencies  (PISA 2006, PISA 2018) ^1^ | Plausible value in math literacy, science literacy, reading literacy; metric variable |
| Mother’s occupation,  Father’s occupation  (PISA 2006, PISA 2018) ^1^ | Categorical variable: “female-dominated” (>= 70% women), “male-dominated” (<= 30% women), and “gender-balanced” (> 30% and < 70% women) occupations; “unknown” all cases with missing information are in a fourth category. |
| ^1^ Student questionnaire | |

| **Table A.2**: Overview of country variables means by country and year | | | | | | | | | | | | | | | | | | | | | | | | | | | | | | | | | | | | | | | |
| --- | --- | --- | --- | --- | --- | --- | --- | --- | --- | --- | --- | --- | --- | --- | --- | --- | --- | --- | --- | --- | --- | --- | --- | --- | --- | --- | --- | --- | --- | --- | --- | --- | --- | --- | --- | --- | --- | --- | --- |
| Country | Women in Employment  (in percentage) | | | | | Women in Parliament  (female-male ratio) | | | | | | Women in Management (rate) | | | | | | Dissimilarity Index | | | | | Gender norms | | | | | Self-Expression | | | | | | GDP per Capita (in Tsd. US$) | | | | | |
|  |  |  | | *A cross years* | |  | |  | | *A cross years* | |  | |  | | *A cross years* | |  | |  | *A cross years* | |  | |  | *A cross years* | |  | |  | *A cross years* | | |  | |  | | *A cross years* | |
|  | 2006 | | 2018 | *mean* | *sd* | 2006 | 2018 | | *mean* | | *sd* | 2010 | 2018 | | *mean* | | *sd* | 2010 | 2018 | | *mean* | *sd* | 2006 | 2018 | | *mean* | *sd* | 2006 | 2018 | | | *mean* | *sd* | 2006 | 2018 | | *mean* | | *sd* |
| Austria | 51.24 | | 55.05 | 53.15 | 2.69 | 0.47 | 0.53 | | 0.50 | | 0.04 | 0.29 | 0.32 | | 0.30 | | 0.02 | 50.28 | 52.45 | | 51.36 | 1.53 | 0.55 | 0.72 | | 0.63 | 0.12 | 4.07 | 4.92 | | | 4.49 | 0.60 | 50.77 | 55.3 | | 53.03 | | 3.20 |
| Belgium | 45.62 | | 48.66 | 47.14 | 2.15 | 0.53 | 0.61 | | 0.57 | | 0.06 | 0.34 | 0.34 | | 0.34 | | 0.00 | 46.79 | 49.35 | | 48.07 | 1.81 | 0.79 | 0.85 | | 0.82 | 0.05 | 3.92 | 4.26 | | | 4.09 | 0.24 | 47.21 | 51.12 | | 49.16 | | 2.76 |
| Bulgaria | 46.88 | | 49.34 | 48.11 | 1.74 | 0.28 | 0.31 | | 0.30 | | 0.02 | 0.33 | 0.39 | | 0.36 | | 0.04 | 47.00 | 53.13 | | 50.06 | 4.33 | 0.79 | 0.89 | | 0.84 | 0.07 | 3.06 | 2.88 | | | 2.97 | 0.13 | 15.45 | 22.21 | | 18.83 | | 4.78 |
| Czech Republic | 50.63 | | 53.05 | 51.84 | 1.71 | 0.18 | 0.28 | | 0.23 | | 0.07 | 0.28 | 0.27 | | 0.27 | | 0.01 | 50.77 | 51.64 | | 51.21 | 0.62 | 0.81 | 0.78 | | 0.80 | 0.02 | 4.28 | 4.75 | | | 4.51 | 0.33 | 32.35 | 39.93 | | 36.14 | | 5.36 |
| Denmark | 61.16 | | 57.45 | 59.31 | 2.62 | 0.58 | 0.60 | | 0.59 | | 0.01 | 0.22 | 0.26 | | 0.24 | | 0.03 | 43.18 | 43.95 | | 43.56 | 0.54 | 0.86 | 0.91 | | 0.89 | 0.03 | 5.17 | 6.51 | | | 5.84 | 0.94 | 53.32 | 56.28 | | 54.80 | | 2.09 |
| Estonia | 54.73 | | 57.38 | 56.06 | 1.87 | 0.23 | 0.36 | | 0.30 | | 0.09 | 0.36 | 0.36 | | 0.36 | | 0.00 | 53.04 | 48.80 | | 50.92 | 3.00 | 0.71 | 0.8 | | 0.75 | 0.07 | 2.96 | 3.73 | | | 3.35 | 0.54 | 28.79 | 35.21 | | 32.00 | | 4.54 |
| Finland | 57.09 | | 55.74 | 56.42 | 0.95 | 0.61 | 0.72 | | 0.67 | | 0.08 | 0.30 | 0.32 | | 0.31 | | 0.01 | 52.41 | 51.17 | | 51.79 | 0.88 | 0.95 | 0.96 | | 0.95 | 0.01 | 4.06 | 5.43 | | | 4.74 | 0.97 | 46.3 | 48.15 | | 47.23 | | 1.31 |
| France | 49.95 | | 50.79 | 50.37 | 0.59 | 0.14 | 0.66 | | 0.40 | | 0.37 | 0.38 | 0.34 | | 0.36 | | 0.03 | 45.97 | 48.72 | | 47.35 | 1.95 | 0.77 | 0.87 | | 0.82 | 0.07 | 4.2 | 5.11 | | | 4.65 | 0.64 | 42.37 | 45.34 | | 43.86 | | 2.10 |
| Germany | 51.47 | | 55.23 | 53.35 | 2.66 | 0.47 | 0.44 | | 0.45 | | 0.02 | 0.30 | 0.29 | | 0.30 | | 0.00 | 48.42 | 47.55 | | 47.98 | 0.61 | 0.67 | 0.73 | | 0.70 | 0.04 | 4.05 | 5.36 | | | 4.70 | 0.93 | 45.61 | 53.46 | | 49.54 | | 5.55 |
| Greece | 43.02 | | 44.39 | 43.70 | 0.97 | 0.15 | 0.23 | | 0.19 | | 0.06 | 0.30 | 0.27 | | 0.29 | | 0.02 | 43.43 | 43.85 | | 43.64 | 0.29 | 0.76 | 0.76 | | 0.76 | 0.00 | 3.93 | 3.31 | | | 3.62 | 0.44 | 36.88 | 29.76 | | 33.32 | | 5.03 |
| Hungary | 43.20 | | 48.45 | 45.83 | 3.71 | 0.12 | 0.14 | | 0.13 | | 0.01 | 0.36 | 0.39 | | 0.38 | | 0.01 | 46.93 | 52.00 | | 49.46 | 3.58 | 0.78 | 0.79 | | 0.78 | 0.01 | 2.3 | 3.54 | | | 2.92 | 0.88 | 25.38 | 31.1 | | 28.24 | | 4.04 |
| Iceland | 71.51 | | 71.06 | 71.28 | 0.32 | 0.50 | 0.62 | | 0.56 | | 0.08 | 0.34 | 0.41 | | 0.38 | | 0.05 | 44.02 | 44.62 | | 44.32 | 0.42 | 0.86 | 0.93 | | 0.89 | 0.05 | 4.73 | 6.01 | | | 5.37 | 0.91 | 48.42 | 55.69 | | 52.05 | | 5.14 |
| Ireland | 55.38 | | 56.15 | 55.77 | 0.54 | 0.15 | 0.28 | | 0.22 | | 0.09 | 0.39 | 0.36 | | 0.38 | | 0.02 | 39.88 | 44.83 | | 42.36 | 3.50 | 0.71 | 0.79 | | 0.75 | 0.06 | 2.96 | 3.35 | | | 3.15 | 0.27 | 57.97 | 83.12 | | 70.55 | | 17.78 |
| Italy | 37.94 | | 40.84 | 39.39 | 2.05 | 0.21 | 0.56 | | 0.38 | | 0.25 | 0.33 | 0.27 | | 0.30 | | 0.04 | 44.80 | 47.47 | | 46.13 | 1.89 | 0.64 | 0.69 | | 0.67 | 0.04 | 3.67 | 4.33 | | | 4.00 | 0.47 | 44.92 | 42.05 | | 43.49 | | 2.03 |
| Latvia | 52.02 | | 56.03 | 54.02 | 2.84 | 0.23 | 0.19 | | 0.21 | | 0.03 | 0.42 | 0.45 | | 0.43 | | 0.02 | 50.73 | 57.49 | | 54.11 | 4.78 | 0.76 | 0.79 | | 0.77 | 0.03 | 2.46 | 2.67 | | | 2.57 | 0.15 | 22.8 | 30.04 | | 26.42 | | 5.12 |
| Lithuania | 50.13 | | 56.73 | 53.43 | 4.67 | 0.28 | 0.27 | | 0.28 | | 0.01 | 0.41 | 0.39 | | 0.40 | | 0.01 | 51.20 | 52.05 | | 51.62 | 0.60 | 0.76 | 0.78 | | 0.77 | 0.01 | 2.53 | 3.06 | | | 2.79 | 0.38 | 22.98 | 35.43 | | 29.20 | | 8.80 |
| Luxembourg | 46.71 | | 54.71 | 50.71 | 5.66 | 0.30 | 0.40 | | 0.35 | | 0.07 | 0.25 | 0.25 | | 0.25 | | 0.00 | 38.50 | 39.42 | | 38.96 | 0.65 | 0.76 | 0.85 | | 0.80 | 0.06 | 4.43 | 4.28 | | | 4.35 | 0.11 | 107.68 | 113.59 | | 110.64 | | 4.18 |
| Netherlands | 55.43 | | 58.37 | 56.90 | 2.08 | 0.58 | 0.56 | | 0.57 | | 0.01 | 0.28 | 0.26 | | 0.27 | | 0.02 | 44.56 | 44.50 | | 44.53 | 0.04 | 0.81 | 0.82 | | 0.82 | 0.01 | 5.15 | 5.82 | | | 5.48 | 0.47 | 51.1 | 56.06 | | 53.58 | | 3.51 |
| Norway | 61.03 | | 60.44 | 60.73 | 0.42 | 0.61 | 0.71 | | 0.66 | | 0.07 | 0.34 | 0.36 | | 0.35 | | 0.01 | 43.59 | 43.54 | | 43.57 | 0.04 | 0.7 | 0.91 | | 0.81 | 0.14 | 3.65 | 5.98 | | | 4.81 | 1.64 | 62.85 | 64.45 | | 63.65 | | 1.13 |
| Poland | 46.70 | | 48.91 | 47.81 | 1.56 | 0.26 | 0.39 | | 0.32 | | 0.09 | 0.36 | 0.43 | | 0.39 | | 0.05 | 49.36 | 52.93 | | 51.15 | 2.52 | 0.54 | 0.65 | | 0.59 | 0.07 | 2.72 | 3.28 | | | 3.00 | 0.39 | 20.11 | 31.67 | | 25.89 | | 8.18 |
| Portugal | 55.58 | | 54.52 | 55.05 | 0.75 | 0.27 | 0.53 | | 0.40 | | 0.18 | 0.32 | 0.34 | | 0.33 | | 0.01 | 45.41 | 48.96 | | 47.19 | 2.51 | 0.67 | 0.78 | | 0.72 | 0.07 | 2.9 | 3.68 | | | 3.29 | 0.55 | 31.52 | 34.04 | | 32.78 | | 1.78 |
| Slovakia | 50.67 | | 52.38 | 51.52 | 1.21 | 0.25 | 0.25 | | 0.25 | | 0.00 | 0.35 | 0.32 | | 0.33 | | 0.02 | 49.99 | 50.42 | | 50.21 | 0.31 | 0.81 | 0.86 | | 0.83 | 0.03 | 3.6 | 4.07 | | | 3.83 | 0.33 | 21.78 | 31.18 | | 26.48 | | 6.65 |
| Slovenia | 53.21 | | 53.81 | 53.51 | 0.42 | 0.14 | 0.32 | | 0.23 | | 0.13 | 0.35 | 0.39 | | 0.37 | | 0.03 | 41.26 | 46.53 | | 43.89 | 3.73 | 0.82 | 0.88 | | 0.85 | 0.04 | 4.28 | 4.73 | | | 4.51 | 0.31 | 32.82 | 37.97 | | 35.39 | | 3.64 |
| Spain | 47.49 | | 52.01 | 49.75 | 3.20 | 0.56 | 0.64 | | 0.23 | | 0.13 | 0.34 | 0.32 | | 0.33 | | 0.02 | 46.46 | 48.90 | | 47.68 | 1.72 | 0.77 | 0.77 | | 0.77 | 0.00 | 3.93 | 5.02 | | | 4.47 | 0.77 | 38.86 | 40.31 | | 39.59 | | 1.03 |
| Sweden | 59.22 | | 61.16 | 60.19 | 1.37 | 0.90 | 0.86 | | 0.88 | | 0.03 | 0.31 | 0.38 | | 0.35 | | 0.05 | 44.15 | 41.21 | | 42.68 | 2.08 | 0.84 | 0.91 | | 0.88 | 0.05 | 5.71 | 6.35 | | | 6.03 | 0.46 | 47.29 | 52.35 | | 49.82 | | 3.58 |
| U. Kingdom | 55.15 | | 57.57 | 56.36 | 1.71 | 0.25 | 0.47 | | 0.36 | | 0.16 | 0.36 | 0.36 | | 0.36 | | 0.00 | 42.49 | 42.32 | | 42.41 | 0.13 | 0.73 | 0.82 | | 0.78 | 0.06 | 4.72 | 5.21 | | | 4.96 | 0.34 | 43.45 | 46.14 | | 44.80 | | 1.90 |
| *A cross countries* | | | | | | | | | | | | | | | | | | | | | | | | | | | | | | | | | | | | | | | |
| *mean* | 52.04 | | 54.24 |  |  | 0.36 | 0.46 | |  | |  | 0.33 | 0.34 | |  | |  | 46.33 | 48.00 | |  |  | 0.75 | 0.82 | |  |  | 3.82 | 4.52 | | |  |  | 41.49 | 47.00 | |  | |  |
| *sd* | 6.90 | | 5.83 |  |  | 0.20 | 0.19 | |  | |  | 0.05 | 0.06 | |  | |  | 3.87 | 4.33 | |  |  | 0.09 | 0.08 | |  |  | 0.89 | 1.12 | | |  |  | 18.46 | 19.00 | |  | |  |

| **Table A.3**: Mean and mean differences in the share of females in aspired occupation by country and year | | | | | | |
| --- | --- | --- | --- | --- | --- | --- |
|  | **Girls** | | | **Boys** | | |
|  | 2006 | 2018 | X^2018^-X^2006^ | 2006 | 2018 | X^2018^-X^2006^ |
| Austria | 0.569 | 0.518 | -0.051 | 0.247 | 0.247 | 0.000 |
| Belgium | 0.588 | 0.504 | -0.084 | 0.259 | 0.283 | 0.025 |
| Bulgaria | 0.603 | 0.654 | 0.051 | 0.587 | 0.536 | -0.051 |
| Czech Republic | 0.574 | 0.588 | 0.014 | 0.267 | 0.262 | -0.006 |
| Denmark | 0.556 | 0.493 | -0.062 | 0.269 | 0.293 | 0.024 |
| Estonia | 0.633 | 0.624 | -0.009 | 0.325 | 0.389 | 0.064 |
| Finland | 0.641 | 0.625 | -0.016 | 0.274 | 0.313 | 0.039 |
| France | 0.533 | 0.484 | -0.048 | 0.271 | 0.282 | 0.011 |
| Germany | 0.577 | 0.518 | -0.059 | 0.302 | 0.285 | -0.017 |
| Greece | 0.465 | 0.462 | -0.003 | 0.287 | 0.301 | 0.014 |
| Hungary | 0.543 | 0.525 | -0.018 | 0.306 | 0.294 | -0.012 |
| Iceland | 0.530 | 0.503 | -0.027 | 0.299 | 0.290 | -0.009 |
| Ireland | 0.589 | 0.549 | -0.040 | 0.267 | 0.331 | 0.065 |
| Italy | 0.442 | 0.455 | 0.014 | 0.258 | 0.279 | 0.021 |
| Latvia | 0.580 | 0.628 | 0.049 | 0.330 | 0.382 | 0.052 |
| Lithuania | 0.640 | 0.652 | 0.011 | 0.370 | 0.338 | -0.032 |
| Luxembourg | 0.514 | 0.472 | -0.042 | 0.257 | 0.292 | 0.035 |
| Netherlands | 0.584 | 0.520 | -0.064 | 0.300 | 0.310 | 0.009 |
| Norway | 0.490 | 0.443 | -0.047 | 0.223 | 0.222 | -0.001 |
| Poland | 0.611 | 0.557 | -0.054 | 0.359 | 0.309 | -0.050 |
| Portugal | 0.541 | 0.463 | -0.078 | 0.309 | 0.295 | -0.015 |
| Slovakia | 0.590 | 0.594 | 0.005 | 0.340 | 0.336 | -0.004 |
| Slovenia | 0.606 | 0.590 | -0.016 | 0.287 | 0.286 | 0.000 |
| Spain | 0.470 | 0.450 | -0.020 | 0.237 | 0.276 | 0.039 |
| Sweden | 0.523 | 0.474 | -0.049 | 0.263 | 0.289 | 0.026 |
| United Kingdom | 0.578 | 0.528 | -0.050 | 0.261 | 0.280 | 0.019 |
| Total | 64,568 | 71,188 |  | 60,917 | 67,887 |  |

| **Table A.4**: Share of female-dominated, male-dominated, and gender-balanced occupational expectations by country, gender, and year | | | | | | | | | | | | | | | | | |
| --- | --- | --- | --- | --- | --- | --- | --- | --- | --- | --- | --- | --- | --- | --- | --- | --- | --- |
|  | Girls | | | | | | |  |  | Boys | | | | | | | |
|  | Female-dominated | | Gender-balanced | | Male-dominated | | N | N |  | Female-dominated | | Gender-balanced | | Male-dominated | | N | N |
|  | 2006 | 2018 | 2006 | 2018 | 2006 | 2018 | 2006 | 2018 | | 2006 | 2018 | 2006 | 2018 | 2006 | 2018 | 2006 | 2018 |
| Austria | 41.50 | 29.44 | 44.08 | 52.62 | 14.41 | 17.94 | 1,665 | 2,347 |  | 5.46 | 4.58 | 33.48 | 32.15 | 61.06 | 63.26 | 1,592 | 2,488 |
| Belgium | 44.09 | 30.82 | 43.31 | 51.61 | 12.60 | 17.58 | 3,302 | 1,337 |  | 9.79 | 9.07 | 29.86 | 36.44 | 60.34 | 54.49 | 3,543 | 1,213 |
| Bulgaria | 18.25 | 28.97 | 69.34 | 67.43 | 12.42 | 3.59 | 1,474 | 1,781 |  | 19.65 | 22.62 | 65.94 | 53.51 | 14.41 | 23.87 | 1,506 | 1,596 |
| Czech Republic | 27.72 | 27.53 | 56.42 | 62.20 | 15.86 | 10.27 | 1,955 | 2,405 |  | 3.62 | 5.29 | 28.26 | 31.05 | 68.13 | 63.66 | 1,964 | 2,229 |
| Denmark | 34.38 | 16.54 | 50.97 | 68.17 | 14.65 | 15.29 | 1,748 | 2,328 |  | 9.20 | 3.82 | 31.12 | 44.78 | 59.68 | 51.40 | 1,674 | 2,175 |
| Estonia | 46.38 | 47.78 | 48.36 | 44.99 | 5.25 | 7.23 | 1,770 | 2,074 |  | 13.27 | 18.65 | 42.98 | 47.27 | 43.75 | 34.08 | 1,703 | 1,957 |
| Finland | 56.17 | 57.72 | 33.33 | 29.01 | 10.50 | 13.26 | 1,896 | 1,968 |  | 15.19 | 14.91 | 24.89 | 33.00 | 59.92 | 52.09 | 1,567 | 1,818 |
| France | 25.99 | 20.01 | 59.41 | 60.03 | 14.60 | 19.69 | 1,774 | 2,184 |  | 4.38 | 4.45 | 36.96 | 38.71 | 58.66 | 56.84 | 1,553 | 2,201 |
| Germany | 40.37 | 25.44 | 45.33 | 55.39 | 14.30 | 19.17 | 1,615 | 1,930 |  | 11.30 | 7.35 | 31.02 | 33.75 | 57.69 | 58.90 | 1,496 | 2,151 |
| Greece | 11.64 | 13.27 | 67.75 | 71.15 | 20.61 | 15.58 | 1,873 | 2,548 |  | 2.82 | 2.91 | 42.79 | 48.18 | 54.39 | 48.91 | 1,526 | 2,339 |
| Hungary | 23.08 | 19.27 | 63.07 | 64.60 | 13.85 | 16.14 | 1,538 | 2,045 |  | 2.75 | 2.32 | 40.91 | 36.25 | 56.35 | 61.43 | 1,457 | 1,986 |
| Iceland | 35.71 | 27.00 | 47.18 | 57.91 | 17.10 | 15.10 | 1,526 | 1,252 |  | 7.48 | 5.97 | 45.28 | 47.71 | 47.24 | 46.32 | 1,323 | 1,155 |
| Ireland | 36.93 | 29.10 | 51.96 | 60.78 | 11.11 | 10.12 | 1,909 | 2,254 |  | 4.70 | 6.61 | 37.69 | 48.31 | 57.61 | 45.08 | 1,767 | 2,074 |
| Italy | 5.30 | 12.93 | 80.19 | 73.29 | 14.51 | 13.78 | 8,449 | 3,983 |  | 0.75 | 2.44 | 44.65 | 46.15 | 54.60 | 51.41 | 8,288 | 3,931 |
| Latvia | 44.89 | 51.33 | 47.43 | 40.95 | 7.68 | 7.71 | 1,887 | 2,061 |  | 12.51 | 15.12 | 51.28 | 53.56 | 36.21 | 31.32 | 1,519 | 1,839 |
| Lithuania | 45.53 | 54.60 | 45.36 | 29.49 | 9.11 | 15.90 | 1,746 | 2,465 |  | 14.15 | 14.44 | 48.04 | 40.34 | 37.80 | 45.22 | 1,611 | 2,417 |
| Luxembourg | 22.41 | 17.36 | 58.48 | 63.43 | 19.11 | 19.21 | 1,821 | 2,051 |  | 5.13 | 6.63 | 36.01 | 41.71 | 58.87 | 51.66 | 1,658 | 1,870 |
| Netherlands | 38.88 | 28.80 | 51.03 | 53.45 | 10.09 | 17.75 | 2,042 | 1,375 |  | 4.57 | 5.54 | 48.96 | 41.44 | 46.47 | 53.02 | 2,012 | 1,192 |
| Norway | 26.50 | 22.02 | 47.74 | 46.78 | 25.76 | 31.20 | 1,638 | 2,112 |  | 2.39 | 3.71 | 28.42 | 26.91 | 69.18 | 69.38 | 1,629 | 2,051 |
| Poland | 48.85 | 34.97 | 39.49 | 50.13 | 11.66 | 14.90 | 2,213 | 2,362 |  | 16.57 | 9.49 | 32.60 | 34.59 | 50.84 | 55.92 | 2,034 | 2,171 |
| Portugal | 18.14 | 4.86 | 63.57 | 70.46 | 18.29 | 24.67 | 2,045 | 2,282 |  | 1.97 | 2.11 | 40.15 | 35.33 | 57.87 | 62.56 | 1,823 | 2,417 |
| Slovakia | 42.91 | 41.28 | 49.40 | 47.06 | 7.69 | 11.66 | 1,678 | 2,161 |  | 14.36 | 10.43 | 47.46 | 48.40 | 38.18 | 41.17 | 1,595 | 1,936 |
| Slovenia | 31.97 | 31.63 | 51.05 | 50.29 | 16.98 | 18.08 | 2,374 | 2,235 |  | 6.56 | 5.64 | 27.60 | 29.73 | 65.84 | 64.63 | 2,529 | 2,516 |
| Spain | 14.39 | 15.65 | 70.93 | 66.19 | 14.68 | 18.16 | 7,316 | 14,400 |  | 0.73 | 2.68 | 39.39 | 43.88 | 59.88 | 53.45 | 6,606 | 13,713 |
| Sweden | 26.10 | 24.33 | 57.85 | 52.22 | 16.05 | 23.45 | 1,751 | 2,047 |  | 3.18 | 6.35 | 39.09 | 34.66 | 57.73 | 58.99 | 1,760 | 1,907 |
| United Kingdom | 36.04 | 26.28 | 55.92 | 62.05 | 8.04 | 11.67 | 5,563 | 5,201 |  | 4.59 | 4.84 | 43.03 | 46.05 | 52.37 | 49.11 | 5,182 | 4,545 |
| Total | 28.73 | 25.62 | 57.74 | 58.44 | 13.52 | 15.94 | 64,568 | 71,188 |  | 6.12 | 6.33 | 39.40 | 41.14 | 54.48 | 52.54 | 60,917 | 67,887 |

|  | | |  | |  | |  | |  | |  | |  | |  |
| --- | --- | --- | --- | --- | --- | --- | --- | --- | --- | --- | --- | --- | --- | --- | --- |
| **Table A.5**: Change in the dependent variable between 2006 and 2018 with controlling for composition effects by state | | | | | | | | | | | | | | | |
|  | *Share of Females* | | | *Female-dominated* | | | | *Male-dominated* | | | | *Gender-balanced* | | | |
| Country | **Girls** | **Boys** | | **Girls** | | **Boys** | | **Girls** | | **Boys** | | **Girls** | | **Boys** | |
| Austria | -0.039*** | 0.002 | | -0.095*** | | -0.006 | | 0.019 | | 0.040** | | 0.076*** | | -0.034* | |
| Belgium | -0.085*** | 0.029*** | | -0.130*** | | 0.002 | | 0.046*** | | -0.061*** | | 0.084*** | | 0.059*** | |
| Bulgaria | 0.054*** | -0.055*** | | 0.105*** | | 0.037** | | -0.091*** | | 0.106*** | | -0.014 | | -0.143*** | |
| Czech Republic | -0.008 | 0.013* | | -0.038*** | | 0.023*** | | -0.043*** | | -0.084*** | | 0.081*** | | 0.061*** | |
| Denmark | -0.047*** | 0.010 | | -0.129*** | | -0.043*** | | 0.012 | | -0.072*** | | 0.118*** | | 0.088*** | |
| Estonia | -0.012 | 0.074*** | | 0.012 | | 0.059*** | | 0.019** | | -0.120*** | | -0.030* | | 0.062*** | |
| Finland | -0.016* | 0.028*** | | 0.010 | | -0.014 | | 0.031*** | | -0.060*** | | -0.041*** | | 0.074*** | |
| France | -0.045*** | 0.013* | | -0.053*** | | 0.001 | | 0.046*** | | -0.024 | | 0.008 | | 0.023 | |
| Germany | -0.073*** | -0.013 | | -0.175*** | | -0.041*** | | 0.052*** | | 0.005 | | 0.123*** | | 0.036** | |
| Greece | -0.001 | 0.011* | | 0.018* | | -0.001 | | -0.050*** | | -0.050*** | | 0.032** | | 0.052*** | |
| Hungary | -0.023*** | -0.011 | | -0.048*** | | -0.005 | | 0.024* | | 0.058*** | | 0.025 | | -0.053*** | |
| Iceland | -0.032*** | -0.013 | | -0.096*** | | -0.017* | | -0.018 | | -0.003 | | 0.114*** | | 0.020 | |
| Ireland | -0.024*** | 0.058*** | | -0.052*** | | 0.020*** | | -0.013 | | -0.105*** | | 0.065*** | | 0.086*** | |
| Italy | 0.011*** | 0.030*** | | 0.074*** | | 0.016*** | | -0.001 | | -0.065*** | | -0.073*** | | 0.050*** | |
| Latvia | 0.035*** | 0.065*** | | 0.039** | | 0.038*** | | 0.007 | | -0.073*** | | -0.046*** | | 0.035* | |
| Lithuania | 0.012 | -0.027*** | | 0.092*** | | 0.006 | | 0.070*** | | 0.069*** | | -0.161*** | | -0.074*** | |
| Luxembourg | -0.037*** | 0.033*** | | -0.048*** | | 0.018** | | 0.007 | | -0.062*** | | 0.040** | | 0.044** | |
| Netherlands | -0.068*** | 0.017** | | -0.105*** | | 0.007 | | 0.077*** | | 0.044** | | 0.027 | | -0.051*** | |
| Norway | -0.033*** | -0.012 | | -0.016 | | 0.010* | | 0.049*** | | 0.017 | | -0.032* | | -0.027* | |
| Poland | -0.052*** | -0.048*** | | -0.128*** | | -0.066*** | | 0.038*** | | 0.050*** | | 0.090*** | | 0.016 | |
| Portugal | -0.073*** | -0.017*** | | -0.122*** | | -0.000 | | 0.051*** | | 0.052*** | | 0.071*** | | -0.051*** | |
| Slovakia | 0.007 | -0.006 | | -0.012 | | -0.048*** | | 0.033*** | | 0.032* | | -0.021 | | 0.017 | |
| Slovenia | -0.006 | -0.011 | | 0.041*** | | 0.001 | | 0.017 | | 0.015 | | -0.058*** | | -0.017 | |
| Spain | -0.032*** | 0.032*** | | 0.010* | | 0.023*** | | 0.058*** | | -0.054*** | | -0.069*** | | 0.032*** | |
| Sweden | -0.031*** | 0.018** | | 0.017 | | 0.032*** | | 0.055*** | | 0.029* | | -0.072*** | | -0.061*** | |
| U. Kingdom | -0.043*** | 0.005 | | -0.075*** | | -0.001 | | 0.045*** | | 0.005 | | 0.031*** | | -0.004 | |
| *** p<0.01, ** p<0.05, * <0.1  Based on linear regressions with individual weights provided by PISA under control of for age, ESCS, migration background, and gender type of father’s and mother’s occupation and clustered standard errors for the schools. | | | | | | | | | | | | | | | |

| **Table A.6** Results of linear regression model of the first level with the metric outcome (share of women in aspired occupation) | | | | |
| --- | --- | --- | --- | --- |
|  | **Girls** | | **Boys** | |
|  | 2006 | 2018 | 2006 | 2018 |
| Age | -0.001 | -0.001 | -0.001 | 0.001 |
| ESCS | -0.023*** | -0.020*** | 0.020*** | 0.014*** |
| *Migration status (Ref. native)* |  |  |  |  |
| 2nd Generation | -0.045*** | -0.036*** | 0.041*** | 0.017*** |
| 1st Generation | -0.050*** | -0.045*** | 0.025*** | 0.020*** |
| *Competencies* |  |  |  |  |
| Mathematics | -0.088*** | -0.134*** | -0.016 | -0.015 |
| Reading | -0.040** | -0.005 | 0.119*** | 0.260*** |
| Science | -0.115*** | -0.185*** | 0.039* | -0.038 |
| *Occupation Mother (Ref. female dominated)* |  |  |  |  |
| gender balanced | -0.017*** | -0.022*** | -0.001 | -0.001 |
| male-dominated | -0.025*** | -0.027*** | -0.008* | -0.004 |
| Missing | -0.022*** | -0.022*** | -0.003 | -0.002 |
| *Occupation father (Ref. female dominated)* |  |  |  |  |
| gender balanced | -0.004 | -0.012** | -0.017*** | -0.016*** |
| male-dominated | -0.001 | -0.011** | -0.051*** | -0.054*** |
| Missing | 0.002 | -0.005 | -0.029*** | -0.032*** |
| *Countries* |  |  |  |  |
| Austria | 0.597*** | 0.384*** | 0.280*** | 0.387*** |
| Belgium | 0.622*** | 0.358*** | 0.289*** | 0.426*** |
| Bulgaria | 0.580*** | 0.478*** | 0.648*** | 0.681*** |
| Czech Republic | 0.611*** | 0.433*** | 0.288*** | 0.399*** |
| Denmark | 0.574*** | 0.352*** | 0.300*** | 0.417*** |
| Estonia | 0.668*** | 0.481*** | 0.343*** | 0.519*** |
| Finland | 0.688*** | 0.483*** | 0.291*** | 0.443*** |
| France | 0.550*** | 0.329*** | 0.302*** | 0.423*** |
| Germany | 0.611*** | 0.376*** | 0.323*** | 0.420*** |
| Greece | 0.472*** | 0.299*** | 0.336*** | 0.450*** |
| Hungary | 0.556*** | 0.361*** | 0.345*** | 0.439*** |
| Iceland | 0.563*** | 0.358*** | 0.325*** | 0.425*** |
| Ireland | 0.610*** | 0.403*** | 0.300*** | 0.462*** |
| Italy | 0.436*** | 0.285*** | 0.314*** | 0.441*** |
| Latvia | 0.598*** | 0.464*** | 0.353*** | 0.524*** |
| Lithuania | 0.653*** | 0.489*** | 0.411*** | 0.480*** |
| Luxembourg | 0.538*** | 0.332*** | 0.284*** | 0.426*** |
| Netherlands | 0.631*** | 0.386*** | 0.321*** | 0.450*** |
| Norway | 0.515*** | 0.306*** | 0.258*** | 0.358*** |
| Poland | 0.625*** | 0.405*** | 0.392*** | 0.448*** |
| Portugal | 0.529*** | 0.300*** | 0.358*** | 0.440*** |
| Slovakia | 0.596*** | 0.429*** | 0.390*** | 0.486*** |
| Slovenia | 0.640*** | 0.464*** | 0.339*** | 0.431*** |
| Spain | 0.501*** | 0.300*** | 0.292*** | 0.422*** |
| Sweden | 0.542*** | 0.337*** | 0.288*** | 0.419*** |
| United Kingdom | 0.594*** | 0.368*** | 0.302*** | 0.414*** |
|  |  |  |  |  |
| N | 64,568 | 71,188 | 60,917 | 67,887 |
| *** p<0.01, ** p<0.05, * <0.1  The results are based on the linear regression model with and clustered standard errors for the schools as a cell mean model for the country variable. In this method, all countries are included in the model and no reference category is used. For this, we used in stata the “.ibn” operator and the “noconstant” option for the regression. | | | | |

| Table A.7 Results of the linear regression model of the first level with the **female-dominated** occupation as the outcome | | | | |  |
| --- | --- | --- | --- | --- | --- |
|  | **Girls** | | **Boys** | | |
|  | 2006 | 2018 | 2006 | 2018 | |
| Age | 0.001 | 0.000 | -0.001 | 0.001 | |
| ESCS | -0.041*** | -0.042*** | 0.000 | -0.002 | |
| *Migration status (Ref. native)* |  |  |  |  | |
| 2nd Generation | -0.088*** | -0.090*** | 0.003 | 0.003 | |
| 1st Generation | -0.106*** | -0.097*** | -0.005 | -0.006 | |
| *Competencies* |  |  |  |  | |
| Mathematics | -0.131*** | -0.228*** | -0.018 | -0.059** | |
| Reading | -0.142*** | -0.223*** | 0.026** | 0.069** | |
| Science | -0.231*** | -0.355*** | -0.002 | -0.044* | |
| *Occupation Mother (Ref. female dominated)* |  |  |  |  | |
| gender balanced | -0.032*** | -0.043*** | -0.009*** | -0.009*** | |
| male-dominated | -0.036*** | -0.043*** | -0.009* | -0.010** | |
| missing | -0.043*** | -0.042*** | -0.012*** | -0.008** | |
| *Occupation father (Ref. female dominated)* |  |  |  |  | |
| gender balanced | 0.001 | -0.018* | -0.044*** | -0.027*** | |
| male-dominated | 0.014 | -0.007 | -0.044*** | -0.032*** | |
| missing | 0.017* | -0.005 | -0.035*** | -0.027*** | |
| *Countries* |  |  |  |  | |
| Austria | 0.456*** | -0.059*** | 0.108*** | 0.065*** | |
| Belgium | 0.493*** | -0.071** | 0.141*** | 0.104*** | |
| Bulgaria | 0.131*** | -0.151*** | 0.240*** | 0.234*** | |
| Czech Republic | 0.350*** | -0.098*** | 0.081*** | 0.064*** | |
| Denmark | 0.363*** | -0.185*** | 0.132*** | 0.049*** | |
| Estonia | 0.522*** | 0.117*** | 0.173*** | 0.197*** | |
| Finland | 0.648*** | 0.219*** | 0.194*** | 0.156*** | |
| France | 0.285*** | -0.193*** | 0.090*** | 0.052*** | |
| Germany | 0.461*** | -0.106*** | 0.162*** | 0.090*** | |
| Greece | 0.125*** | -0.278*** | 0.081*** | 0.040*** | |
| Hungary | 0.252*** | -0.214*** | 0.074*** | 0.034*** | |
| Iceland | 0.407*** | -0.105*** | 0.124*** | 0.071*** | |
| Ireland | 0.403*** | -0.076*** | 0.094*** | 0.076*** | |
| Italy | 0.047*** | -0.287*** | 0.057*** | 0.034*** | |
| Latvia | 0.477*** | 0.102*** | 0.163*** | 0.165*** | |
| Lithuania | 0.468*** | 0.138*** | 0.185*** | 0.150*** | |
| Luxembourg | 0.261*** | -0.186*** | 0.099*** | 0.079*** | |
| Netherlands | 0.464*** | -0.067*** | 0.096*** | 0.071*** | |
| Norway | 0.300*** | -0.133*** | 0.072*** | 0.049*** | |
| Poland | 0.513*** | -0.033* | 0.210*** | 0.108*** | |
| Portugal | 0.156*** | -0.356*** | 0.066*** | 0.030*** | |
| Slovakia | 0.428*** | -0.009 | 0.199*** | 0.117*** | |
| Slovenia | 0.344*** | -0.026 | 0.111*** | 0.076*** | |
| Spain | 0.184*** | -0.220*** | 0.061*** | 0.041*** | |
| Sweden | 0.289*** | -0.109*** | 0.075*** | 0.073*** | |
| United Kingdom | 0.381*** | -0.125*** | 0.091*** | 0.053*** | |
|  |  |  |  |  | |
| N | 64,568 | 71,188 | 60,917 | 67,887 | |
| *** p<0.01, ** p<0.05, * <0.1  The results are based on the linear regression model with and clustered standard errors for the schools as a cell mean model for the country variable. In this method, all countries are included in the model and no reference category is used. For this, we used in stata the “.ibn” operator and the “noconstant” option for the regression. | | | | | |

| **Table A.8** Results of linear regression model of the first level with the **male-dominated** occupation as the outcome | | | | |  |
| --- | --- | --- | --- | --- | --- |
|  | **Girls** | | **Boys** | | |
|  | 2006 | 2018 | 2006 | 2018 | |
| Age | 0.000 | 0.003* | -0.001 | -0.002 | |
| ESCS | 0.004** | 0.008*** | -0.047*** | -0.030*** | |
| *Migration status (Ref. native)* |  |  |  |  | |
| 2nd Generation | -0.002 | -0.009 | -0.112*** | -0.029*** | |
| 1st Generation | 0.005 | -0.001 | -0.070*** | -0.039*** | |
| *Competencies* |  |  |  |  | |
| Mathematics | 0.100*** | 0.139*** | 0.088* | 0.0960 | |
| Reading | -0.039* | -0.187*** | -0.245*** | -0.554*** | |
| Science | 0.0260 | 0.109** | -0.077* | 0.160** | |
| *Occupation Mother (Ref. female dominated)* |  |  |  |  | |
| gender balanced | 0.007* | 0.014*** | -0.011* | -0.017*** | |
| male-dominated | 0.028*** | 0.033*** | 0.013 | 0.011 | |
| missing | 0.014*** | 0.024*** | -0.001 | -0.003 | |
| *Occupation father (Ref. female dominated)* |  |  |  |  | |
| gender balanced | 0.003 | 0.002 | -0.021* | -0.005 | |
| male dominated | 0.021*** | 0.020** | 0.083*** | 0.101*** | |
| missing | 0.011 | 0.006 | 0.034** | 0.050*** | |
| *Countries* |  |  |  |  | |
| Austria | 0.125*** | 0.202*** | 0.581*** | 0.457*** | |
| Belgium | 0.097*** | 0.189*** | 0.569*** | 0.341*** | |
| Bulgaria | 0.126*** | 0.069*** | 0.065*** | 0.053** | |
| Czech Republic | 0.140*** | 0.139*** | 0.679*** | 0.447*** | |
| Denmark | 0.125*** | 0.181*** | 0.566*** | 0.355*** | |
| Estonia | 0.033** | 0.099*** | 0.443*** | 0.166*** | |
| Finland | 0.079*** | 0.162*** | 0.590*** | 0.347*** | |
| France | 0.129*** | 0.220*** | 0.561*** | 0.375*** | |
| Germany | 0.124*** | 0.215*** | 0.571*** | 0.405*** | |
| Greece | 0.195*** | 0.187*** | 0.486*** | 0.290*** | |
| Hungary | 0.123*** | 0.193*** | 0.515*** | 0.419*** | |
| Iceland | 0.148*** | 0.172*** | 0.459*** | 0.287*** | |
| Ireland | 0.092*** | 0.131*** | 0.539*** | 0.280*** | |
| Italy | 0.139*** | 0.176*** | 0.468*** | 0.264*** | |
| Latvia | 0.062*** | 0.110*** | 0.352*** | 0.122*** | |
| Lithuania | 0.073*** | 0.187*** | 0.328*** | 0.258*** | |
| Luxembourg | 0.176*** | 0.222*** | 0.578*** | 0.342*** | |
| Netherlands | 0.069*** | 0.189*** | 0.464*** | 0.333*** | |
| Norway | 0.236*** | 0.333*** | 0.662*** | 0.514*** | |
| Poland | 0.100*** | 0.179*** | 0.477*** | 0.369*** | |
| Portugal | 0.178*** | 0.267*** | 0.514*** | 0.421*** | |
| Slovakia | 0.064*** | 0.142*** | 0.316*** | 0.206*** | |
| Slovenia | 0.128*** | 0.185*** | 0.585*** | 0.446*** | |
| Spain | 0.099*** | 0.204*** | 0.527*** | 0.333*** | |
| Sweden | 0.150*** | 0.256*** | 0.564*** | 0.417*** | |
| United Kingdom | 0.054*** | 0.141*** | 0.461*** | 0.293*** | |
|  |  |  |  |  | |
| N | 64,568 | 71,188 | 60,917 | 67,887 | |
| *** p<0.01, ** p<0.05, * <0.1  The results are based on the linear regression model with and clustered standard errors for the schools as a cell mean model for the country variable. In this method, all countries are included in the model and no reference category is used. For this we used in stata the “.ibn” operator and the “noconstant” option for the regression. | | | | | |

| **Table A.9** Results of linear regression model of the first level with the **gender-balanced** occupation as the outcome | | | | |  |
| --- | --- | --- | --- | --- | --- |
|  | **Girls** | | **Boys** | | |
|  | 2006 | 2018 | 2006 | 2018 | |
| Age | -0.001 | -0.004* | 0.002 | 0.001 | |
| ESCS | 0.037*** | 0.034*** | 0.048*** | 0.032*** | |
| *Migration status (Ref. native)* |  |  |  |  | |
| 2nd Generation | 0.091*** | 0.099*** | 0.109*** | 0.026*** | |
| 1st Generation | 0.101*** | 0.098*** | 0.075*** | 0.046*** | |
| *Competencies* |  |  |  |  | |
| Mathematics | 0.031 | 0.089* | -0.070* | -0.037 | |
| Reading | 0.181*** | 0.410*** | 0.219*** | 0.485*** | |
| Science | 0.205*** | 0.246*** | 0.078* | -0.116* | |
| *Occupation Mother (Ref. female dominated)* |  |  |  |  | |
| gender balanced | 0.026*** | 0.029*** | 0.019*** | 0.026*** | |
| male-dominated | 0.008 | 0.010 | -0.004 | 0.000 | |
| missing | 0.029*** | 0.018** | 0.014* | 0.011* | |
| *Occupation father (Ref. female dominated)* |  |  |  |  | |
| gender balanced | -0.004 | 0.016 | 0.065*** | 0.033*** | |
| male-dominated | -0.035*** | -0.013 | -0.038*** | -0.069*** | |
| missing | -0.028** | -0.001 | 0.001 | -0.023* | |
| *Countries* |  |  |  |  | |
| Austria | 0.419*** | 0.857*** | 0.311*** | 0.478*** | |
| Belgium | 0.410*** | 0.882*** | 0.290*** | 0.555*** | |
| Bulgaria | 0.743*** | 1.082*** | 0.694*** | 0.713*** | |
| Czech Republic | 0.510*** | 0.959*** | 0.240*** | 0.488*** | |
| Denmark | 0.512*** | 1.004*** | 0.302*** | 0.596*** | |
| Estonia | 0.445*** | 0.784*** | 0.384*** | 0.637*** | |
| Finland | 0.273*** | 0.619*** | 0.217*** | 0.496*** | |
| France | 0.587*** | 0.972*** | 0.349*** | 0.573*** | |
| Germany | 0.414*** | 0.891*** | 0.268*** | 0.505*** | |
| Greece | 0.680*** | 1.091*** | 0.433*** | 0.670*** | |
| Hungary | 0.625*** | 1.021*** | 0.411*** | 0.547*** | |
| Iceland | 0.445*** | 0.933*** | 0.417*** | 0.642*** | |
| Ireland | 0.506*** | 0.945*** | 0.368*** | 0.644*** | |
| Italy | 0.814*** | 1.112*** | 0.475*** | 0.702*** | |
| Latvia | 0.461*** | 0.788*** | 0.486*** | 0.713*** | |
| Lithuania | 0.459*** | 0.675*** | 0.487*** | 0.592*** | |
| Luxembourg | 0.563*** | 0.964*** | 0.323*** | 0.580*** | |
| Netherlands | 0.467*** | 0.878*** | 0.440*** | 0.596*** | |
| Norway | 0.463*** | 0.800*** | 0.266*** | 0.437*** | |
| Poland | 0.387*** | 0.854*** | 0.313*** | 0.523*** | |
| Portugal | 0.666*** | 1.089*** | 0.419*** | 0.550*** | |
| Slovakia | 0.508*** | 0.867*** | 0.486*** | 0.676*** | |
| Slovenia | 0.528*** | 0.841*** | 0.303*** | 0.478*** | |
| Spain | 0.717*** | 1.016*** | 0.412*** | 0.626*** | |
| Sweden | 0.561*** | 0.853*** | 0.360*** | 0.510*** | |
| United Kingdom | 0.565*** | 0.985*** | 0.448*** | 0.653*** | |
|  |  |  |  |  | |
| N | 64,568 | 71,188 | 60,917 | 67,887 | |
| *** p<0.01, ** p<0.05, * <0.1  The results are based on the linear regression model with and clustered standard errors for the schools as a cell mean model for the country variable. In this method, all countries are included in the model and no reference category is used. For this, we used in stata the “.ibn” operator and the “noconstant” option for the regression. | | | | | |

| **Table A.10**: Change in the dependent variable between 2006 and 2018 | | | | | | | | |
| --- | --- | --- | --- | --- | --- | --- | --- | --- |
|  | *Share of Females* | | *Female-dominated* | | *Male-dominated* | | *Gender-balanced* | |
|  | **Girls** | **Boys** | **Girls** | **Boys** | **Girls** | **Boys** | **Girls** | **Boys** |
| *Ref. 2006*  2018 | -0.192*** | 0.112*** | -0.441*** | -0.035*** | 0.059*** | -0.165*** | 0.382*** | 0.200*** |
| Constant | 0.581*** | 0.333*** | 0.354*** | 0.122*** | 0.118*** | 0.497*** | 0.527*** | 0.381*** |
|  |  |  |  |  |  |  |  |  |
| Observations | 52 | 52 | 52 | 52 | 52 | 52 | 52 | 52 |
| \| *** p<0.01, ** p<0.05, * <0.1 \|  \|  \|  \|  \|  \|  \|  \| \| --- \| --- \| --- \| --- \| --- \| --- \| --- \| --- \| | | | | | | | | |

| **Table A.11**: Amount of significant coefficient of 26 regression model sets excluded one country from the analyses at a time | | | | | | | | | | | | | | | | |
| --- | --- | --- | --- | --- | --- | --- | --- | --- | --- | --- | --- | --- | --- | --- | --- | --- |
|  | | *Share of Females* | | | *Female-dominated* | | | | *Male-dominated* | | | | *Gender-balanced* | | | |
|  | | **Girls** | | **Boys** | **Girls** | | **Boys** | | **Girls** | | **Boys** | | **Girls** | | **Boys** |  |
| *Women’s Empowerment* | |  | |  |  | |  | |  | |  | |  | |  |  |
| Female Employment Ratio | | **26** | | **26** | **26** | | 0 | | **26** | | **24** | | **24** | | **22** |  |
| Women in Parliament | | **25** | | **22** | **25** | | 0 | | 0 | | **25** | | **25** | | **25** |  |
| Women in Management | | 0 | | 0 | 0 | | 0 | | 0 | | 0 | | 2 | | 0 |  |
| Dissimilarity Index | | 0 | | 0 | 0 | | 0 | | 0 | | 1 | | 0 | | 0 |  |
| *Cultural norms and values* | |  | |  |  | |  | |  | |  | |  | |  |  |
| Gender norms | | 0 | | 0 | 0 | | 0 | | 0 | | 0 | | 0 | | 0 |  |
| Self-Expression | | **26** | | **26** | **26** | | **24** | | **26** | | **24** | | **26** | | **26** |  |
| *Economic Development* | |  | |  |  | |  | |  | |  | |  | |  |  |
| GDP | | 6 | | 4 | 7 | | 0 | | 0 | | 5 | | 2 | | 17 |  |
|  | | | | | | | | | | | | | | | | |
| **Table A.12**: Correlation between state characteristics 2006 | | | | | | | | | | | | | | |  |  |
|  | | Female Emplo. | | Female Parliament | | | Female Management | | Dissimilarity Index | | Gender norms | | Self-Expression | |  |  |
| Female Parliament | | 0.494* | | 1.000 | | |  | |  | |  | |  | |  |  |
| Female Management | | -0.089 | | -0.396** | | | 1.000 | |  | |  | |  | |  |  |
| Dissimilarity Index | | -0.116 | | -0.008 | | | 0.239 | | 1.000 | |  | |  | |  |  |
| Gender norms | | 0.370* | | 0.279 | | | -0.160 | | -0.083 | | 1.000 | |  | |  |  |
| Self-Expression | | 0.413** | | 0.589*** | | | -0.637*** | | -0.409** | | 0.408** | | 1.000 | |  |  |
| GDP in tsd | | 0.184 | | 0.324 | | | -0.511*** | | -0.615*** | | 0.049 | | 0.503*** | |  |  |
| *** p<0.01, ** p<0.05, * <0.1 | | | | | | | | | | | | | | |  |  |
| **Table A.13**: Correlation between state characteristics 2018 | | | | | | | | | | | | | | |  |  |
|  | | Female Emplo. | | Female Parliament | | | Female Management | | Dissimilarity Index | | Gender norms | | Self-Expression | |  |  |
| Female Parliament | | 0.338 | | 1.000 | | |  | |  | |  | |  | |  |  |
| Female Management | | 0.268 | | -0.167 | | | 1.000 | |  | |  | |  | |  |  |
| Dissimilarity Index | | -0.312 | | -0.410** | | | 0.413** | | 1.000 | |  | |  | |  |  |
| Gender norms | | 0.519*** | | 0.401** | | | 0.060 | | -0.330 | | 1.000 | |  | |  |  |
| Self-Expression | | 0.519*** | | 0.776*** | | | -0.346* | | -0.566*** | | 0.485** | | 1.000 | |  |  |
| GDP in tsd | | 0.340* | | 0.286 | | | -0.391** | | -0.647*** | | 0.211 | | 0.362* | |  |  |
| *** p<0.01, ** p<0.05, * <0.1 | | | | | | | | | | | | | | |  |  |

| **Table A.14**: Results of the regression model with fixed effects by gender and factors | | | | | | | | |
| --- | --- | --- | --- | --- | --- | --- | --- | --- |
|  | *Share of Females* | | *Female-dominated* | | *Male-dominated* | | *Gender-balanced* | |
|  | **Girls** | **Boys** | **Girls** | **Boys** | **Girls** | **Boys** | **Girls** | **Boys** |
|  |  |  |  |  |  |  |  |  |
| **Female Emplo.** | -0.037*** | 0.023*** | -0.085*** | -0.005** | 0.013*** | -0.032*** | 0.072*** | 0.037*** |
| Constant | 2.465*** | -0.812*** | 4.631*** | 0.391*** | -0.527*** | 2.104*** | -3.105*** | -1.495*** |
| $\hat{\sigma}$ | 0.076 | 0.044 | 0.176 | 0.019 | 0.027 | 0.070 | 0.154 | 0.084 |
| Average ω | 0.007 | 0.007 | 0.014 | 0.008 | 0.012 | 0.016 | 0.016 | 0.016 |
|  |  |  |  |  |  |  |  |  |
| **Female Parliament** | -0.780*** | 0.447*** | -1.748*** | -0.136*** | 0.229*** | -0.706*** | 1.519*** | 0.842*** |
| Constant | 0.802*** | 0.207*** | 0.846*** | 0.160*** | 0.054** | 0.702*** | 0.100 | 0.138 |
| $\hat{\sigma}$ | 0.075 | 0.045 | 0.174 | 0.018 | 0.029 | 0.066 | 0.151 | 0.079 |
| Average ω | 0.007 | 0.007 | 0.014 | 0.008 | 0.012 | 0.016 | 0.016 | 0.016 |
|  |  |  |  |  |  |  |  |  |
| **Women in Management** | -1.149 | 0.369 | -2.900 | -0.336 | 0.207 | -0.455 | 2.694 | 0.792 |
| Constant | 0.871*** | 0.265 | 1.108 | 0.218** | 0.077 | 0.567* | -0.186 | 0.215 |
| $\hat{\sigma}$ | 0.096 | 0.058 | 0.219 | 0.020 | 0.034 | 0.087 | 0.189 | 0.104 |
| Average ω | 0.007 | 0.007 | 0.014 | 0.008 | 0.012 | 0.016 | 0.016 | 0.016 |
|  |  |  |  |  |  |  |  |  |
| **Dissimilarity Index** | -0.030*** | 0.017*** | -0.072*** | -0.006*** | 0.006 | -0.023** | 0.066*** | 0.030*** |
| Constant | 1.894*** | -0.395 | 3.547*** | 0.406*** | -0.155 | 1.520*** | -2.394*** | -0.925* |
| $\hat{\sigma}$ | 0.086 | 0.052 | 0.195 | 0.018 | 0.033 | 0.080 | 0.166 | 0.093 |
| Average ω | 0.007 | 0.007 | 0.014 | 0.008 | 0.012 | 0.016 | 0.016 | 0.016 |
|  |  |  |  |  |  |  |  |  |
| **Gender norms** | -1.886*** | 1.027*** | -4.310*** | -0.339*** | 0.595*** | -1.495*** | 3.716*** | 1.833*** |
| Constant | 1.968*** | -0.419*** | 3.524*** | 0.371*** | -0.321*** | 1.590*** | -2.204*** | -0.960*** |
| $\hat{\sigma}$ | 0.060 | 0.040 | 0.138 | 0.016 | 0.024 | 0.063 | 0.121 | 0.072 |
| Average ω | 0.007 | 0.007 | 0.014 | 0.008 | 0.012 | 0.016 | 0.016 | 0.016 |
|  |  |  |  |  |  |  |  |  |
| **Self-Expression** | -0.173*** | 0.095*** | -0.390*** | -0.034*** | 0.064*** | -0.141*** | 0.326*** | 0.174*** |
| Constant | 1.205*** | -0.007 | 1.763*** | 0.245*** | -0.121*** | 1.002*** | -0.644** | -0.247* |
| \| $\hat{\sigma}$ \| \| --- \| | 0.058 | 0.039 | 0.137 | 0.014 | 0.017 | 0.060 | 0.126 | 0.067 |
| Average ω | 0.007 | 0.007 | 0.014 | 0.008 | 0.012 | 0.016 | 0.016 | 0.016 |
|  |  |  |  |  |  |  |  |  |
| **GDP in tsd** | -0.017*** | 0.010*** | -0.040*** | -0.003** | 0.005*** | -0.015*** | 0.035*** | 0.018*** |
| Constant | 1.244*** | -0.052 | 1.911*** | 0.247*** | -0.091 | 1.062*** | -0.820** | -0.309* |
| $\hat{\sigma}$ | 0.071 | 0.043 | 0.159 | 0.017 | 0.027 | 0.066 | 0.138 | 0.077 |
| Average ω | 0.007 | 0.007 | 0.014 | 0.008 | 0.012 | 0.016 | 0.016 | 0.016 |
|  |  |  |  |  |  |  |  |  |
| Observations | 52 | 52 | 52 | 52 | 52 | 52 | 52 | 52 |
| *** p<0.01, ** p<0.05, * <0.1 | | | | | | | | |
